# Borocarbonitride‐Based Emerging Materials for Supercapacitor Applications: Recent Advances, Challenges, and Future Perspectives

**DOI:** 10.1002/advs.202305325

**Published:** 2023-11-27

**Authors:** Sithara Radhakrishnan, Abhinandan Patra, G. Manasa, Mohammed Arkham Belgami, Sang Mun Jeong, Chandra Sekhar Rout

**Affiliations:** ^1^ Centre for Nano and Material Sciences Jain (Deemed‐to‐be University) Jain Global Campus, Kanakapura Road Bangalore Karnataka 562112 India; ^2^ Department of Chemical Engineering Chungbuk National University Cheongju Chungbuk 28644 Republic of Korea

**Keywords:** 2D materials, charge storage mechanism, composites, electrochemical energy storage, supercapacitors

## Abstract

Supercapacitors have emerged as a promising energy storage technology due to their high‐power density, fast charging/discharging capabilities, and long cycle life. Moreover, innovative electrode materials are extensively explored to enhance the performance, mainly the energy density of supercapacitors. Among the two‐dimensional (2D) supercapacitor electrodes, borocarbonitride (BCN) has sparked widespread curiosity owing to its exceptional tunable properties concerning the change in concentration of the constituent elements, along with an excellent alternative to graphene‐based electrodes. BCN, an advanced nanomaterial, possesses excellent electrical conductivity, chemical stability, and a large specific surface area. These factors contribute to supercapacitors' overall performance and reliability, making them a viable option to address the energy crisis. This review provides a detailed survey of BCN, its structural, electronic, chemical, magnetic, and mechanical properties, advanced synthesis methods, factors affecting the charge storage mechanism, and recent advances in BCN‐based supercapacitor electrodes. The review embarks on the scrupulous elaboration of ways to enhance the electrochemical properties of BCN through various innovative strategies followed by critical challenges and future perspectives. BCN, as an eminent electrode material, holds great potential to revolutionize the energy landscape and support the growing energy demands of the future.

## Graphical abstract

The review article embarks on the scrupulous elaboration of ways to enhance the electrochemical properties of borocarbonitride (BCN) through various innovative strategies followed by critical challenges and future perspectives. BCN as an eminent electrode material holds great potential to revolutionize the energy landscape and support the growing energy demands of the future.

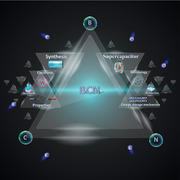

## 1 Introduction

Considering the current status of the world, with a population of ≈7 billion individuals projected to ascend to 10 billion by the year 2050,^[^ ^1^ ^]^ a formidable challenge emerges for the realms of technology and science. Notably, the widespread utilization of fossil fuels as a primary energy source elicits substantial detrimental consequences on the environment while simultaneously depleting the finite reservoirs of natural fuel resources.^[^ ^2^ ^]^ The prevailing circumstances discussed above have initiated a considerable upswing in scientific momentum, specifically within the realm of energy research aimed at advancing sustainable methodologies and devices.^[^ ^3^, ^4^, ^5^, ^6^ ^]^ The overarching goal is to facilitate the efficient conversion of energy into various forms, guided by renewable principles, guaranteeing long‐term viability and mitigating adverse environmental effects. As a result, the pressing need for proficient energy management and storage has acted as a pivotal motivator for researchers to actively pursue the design and optimization of purpose‐built devices to cater to energy storage applications, which resulted in the development of rechargeable batteries and supercapacitors.^[^ ^7^, ^8^, ^9^, ^10^, ^11^ ^]^ There are concerns about the ready‐to‐go option batteries related to their slow ion mobility, short cycling lifespan, and thermal instability.^[^ ^12^ ^]^ In contrast, supercapacitors are emerging as promising energy storage devices attributed to their high‐power density, prolonged life cycle, wide working temperature range, and quick charging and discharging properties. Their nature of feasible combination with the energy conversion systems makes them a great companion for electrical applications, be it in hybrid vehicles or any energy harvesting system.^[^ ^13^, ^14^, ^15^ ^]^ The enigma with supercapacitors lies with their low energy density (≈5 Wh kg^−1^) and affordability.^[^ ^16^ ^]^

The shortcomings of a supercapacitor could be addressed by exploring various electrode material and their performance tuning through the electrolyte and electrode material used. To prioritize the electrode material, the literature depicts numerous electrode materials like carbon‐based graphene, transition metal dichalcogenides (TMDs), nitrides of carbon, metal–organic frameworks, metal sulfides, metal nitrides, etc., to have been explored.^[^ ^8^, ^17^, ^18^, ^19^, ^20^, ^21^ ^]^ One among them is graphene, the 2D material having a sp^2^‐hybridized monolayer of carbon that is the most researched. Despite its several unique traits, the demerit of graphene is the restacking of successive layers that decreases the available surface area, disrupts the structure, and diminishes the charge storage capacity compared to monolayer graphene.^[^ ^21^ ^]^ However, the non‐covalent and covalent functionalization methods have been proven to be the best alternative to improve the properties of graphene. Graphene has been explored in the doped form with N and B. Their prepared composites, nitrides,^[^ ^22^, ^23^, ^24^, ^25^, ^26^ ^]^ and borides^[^ ^27^, ^28^, ^29^, ^30^, ^31^, ^32^ ^]^ have unveiled high theoretical and experimental specific capacitance. The hexagonal boron nitride (h‐BN) has followed a line of investigation in its composite form with various metals, materials like poly‐aniline, MXenes, etc.^[^ ^33^, ^34^, ^35^ ^]^ Likewise, a hybrid of ternary compounds containing carbon (graphene), nitrogen, and boron that can collectively give a higher standard of capacitance and other electrochemical parameters has gained considerable interest over the past few years. This ternary compound is expected to tie the knots of advantages regarding the electrochemical properties of pristine elements in composite form.^[^ ^36^, ^37^ ^]^ It offers plausible applications in optoelectronics, sensing devices, catalysts, lubricants, supercapacitors, etc.^[^ ^38^, ^39^, ^40^, ^41^ ^]^ One such ternary nanomaterial is boron‐carbon‐nitrides (BCN), gaining significant research interest, attributed to its promising traits such as:^[^ ^42^ ^]^ i) versatility (tuneable structure and composition), ii) thermal and electrical conductivity, iii) chemical stability, iv) excellent mechanical properties, and v) wide bandgap semiconducting applications. Another significant trait is its environmental friendliness and sustainability as it is composed of earth‐abundant elements boron (B), carbon (C) and nitrogen (N), which diminishes the dependency on expensive and limited resources. These merits make BCN‐based supercapacitors a compelling choice for energy storage applications, offering reliability, amplified performance, and sustainability.

**Figure 1:**
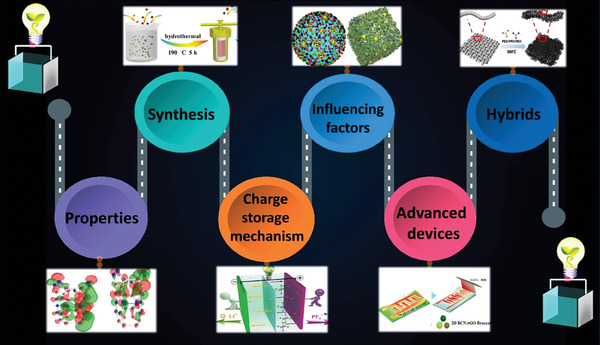
An overview of the BCN‐based supercapacitor devices: structure, property, mechanism, and recent advances.

Although few articles have recently appeared on the synthesis and application of BCN, there are hardly any comprehensive reviews covering the BCN application for supercapacitors in particular.^[^ ^42^, ^43^, ^44^, ^45^, ^46^, ^47^ ^]^ BCN is currently being widely researched concerning energy storage and conversion. For example, combining BCN with transition metal dichalcogens (TMDs), MXene, and polymers improves electrochemical performance. As a result, the synthesis of BCN with relevant compounds must be carefully reviewed, which has not yet been done. This paper focuses on the effect of anode fabrication on essential performance parameters such as capacitance, energy density, and power density in supercapacitors. It also looks at how to increase supercapacitor performance through appropriate capacitor assembly and the impact of electrolytes. The review will focus on the importance of BCN morphology and surface chemistry in electrochemical performance. Furthermore, it also focuses on different anode architectures, notably in pseudo‐capacitor applications. In addition, we will review recent studies to shed light on potential future choices for designing and building BCN electrodes to develop supercapacitors. Initially, we introduce to our readers the BCN and its emerging application in Section 1. Section 2 delves into a detailed discussion of the structure and properties of BCN. Section 3 substantiates the methods of BCN synthesis followed by its application in supercapacitors and the mechanism of charge storage as part of Section 4. While Section 5 provides an overview of recent advances in BCN‐based supercapacitor devices. This review article on BCNs for supercapacitor applications sheds light on the properties, synthesis methodologies, and performance evaluation of BCNs (**Figure** **1**). Finally, Section 6 provides a comprehensive conclusion of the review and the prospects with crucial advice for designing and synthesizing BCN nanostructures to spark more significant applications for supercapacitors.

## 2 Overview of BCN and Its Properties

BCN, having a general formula B_x_C_y_N_z,_ is an emerging class of 2D materials where the composition can be varied over an extensive range. The presence of BN and graphene domains, as well as BCN rings, distinguishes B_x_C_y_N_z_ from B,N co‐doped carbon in terms of structural, compositional, and thermal stability.^[^ ^48^ ^]^ When the stochiometric ratio of BN to carbon is 1:1, the compound is called BCN.^[^ ^49^ ^]^

The elements boron (B‐IIIA group), carbon (C‐IVA group), and nitrogen (N‐VA group) have comparable atomic sizes. Carbon polymorphs and boron nitride (BN) structures are similar, providing a foundation for synthesizing a diamond‐like phase containing all three elements (B–C–N). The B–C–N class is divided into i) B–N and graphene domain‐constituting composites and ii) B–C and N–C bonds coupled with C–C and B–N bonds. The two factors that determine these materials' qualities are the percentage of these bonds and the synthesis procedure. BCN displays tunable electrical and physical characteristics distinct from hexagonal BN (h‐BN).^[^ ^47^, ^50^ ^]^ BCN materials are anticipated to combine the characteristics of graphene, BN, B_4_C, and carbon nitride (C_3_N_4_). The characteristics of B_x_C_y_N_z_ are the consequences of the variations in the combination of B, C, and N atoms. Researchers Lee et al. have reported BCN's hybrid phases, namely c‐B_x_C_y_N_z_, h‐B_x_C_y_N_z_, and nanohybrids with carbon nanotubes (CNT) and boron nitride nanotubes (BNNTs).^[^ ^51^ ^]^ The ternary BCN is unlike the B, N co‐doped graphene or carbon‐doped BN in that it constitutes h‐BCN rings along with graphene and BN domains.^[^ ^42^ ^]^ Thus, structural topography and composition are of fundamental importance in defining the material's properties. Therefore, once the desired material qualities are established, applications that benefit from them are implemented. BCN is among the distinct materials, attributed to its ability to be tuned utilizing various synthesis approaches.^[^ ^52^ ^]^ This has gathered overwhelming attention from both industrial and academic scientists, which gives BCN new opportunities in the fields of electrical (field‐effect transistors (FET), supercapacitors, and high voltage photodetectors), mechanical (good cutting tool, antiwear protective coating), optical (UV detector), adsorption (water purification), nano‐medicine and sensor applications. Several published articles in the literature can be referred to for a detailed description of the diverse utility of BCNs.^[^ ^43^, ^53^, ^54^ ^]^ Moving on, let's discuss the value of BCN toward the nanodevice application, such as the supercapacitor.

Supercapacitors, or electrochemical capacitors, are energy storage devices that rapidly store and deliver energy. They fill the void between batteries (which store energy through chemical reactions) and conventional capacitors (which store energy electrostatically), as they offer fast charging and discharging rates, high power density, and long cycle life.^[^ ^55^, ^56^ ^]^ BCNs exhibit several characteristics that make them promising materials for supercapacitors, including:^[^ ^50^ ^]^ i) Tunable traits: the structure and composition of BCNs can be tailored by adjusting the B, C, and N content along with the synthesis conditions. This enables the optimization of voltage and capacitance ranges. ii) Chemical stability: BCNs are chemically stable, which enables the device to operate reliably in a wide range of electrolytes, minimizing performance degradation over time. They can withstand elevated temperatures without significant performance degradation, ensuring the long‐term reliability of the device. iii) Good electrical conductivity: attributed to their graphitic carbon domains within their structure, they facilitate efficient transfer of charge within the material during charging and discharging cycles (ensures high power delivery capabilities). iv) High surface area: attributed to their porous structure and incorporation of heteroatom, BCNs possess a greater specific surface area that enhances the electrochemical reaction of the material (thus enabling efficient charge storage). v) Wide potential window: BCNs exhibit a broad electrochemical stability window. This trait enables its application in high‐voltage supercapacitor devices, increasing their energy storage capacity.

### 2.1 Structural Attributes

**Figure 2:**
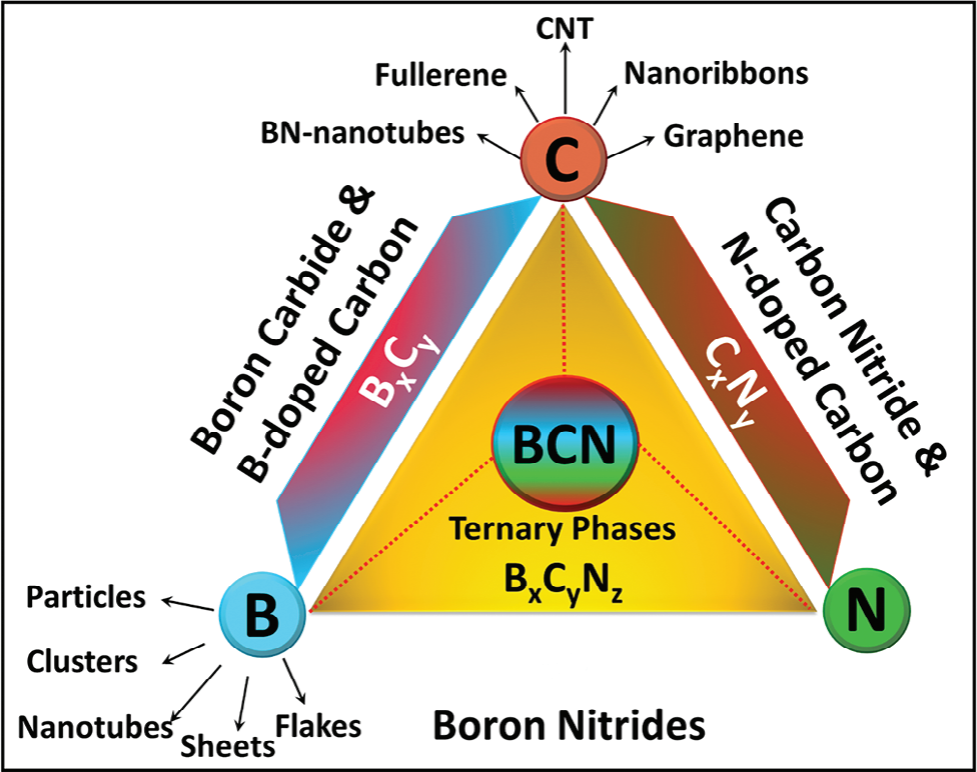
Ternary phase diagram of the BCN system.

B‐C‐N is a novel semiconductor material with excellent chemical and thermal stability of h‐BN.^[^ ^57^ ^]^ Typically, ternary BCN semiconductors possess C atoms (conjugated sp^2^ hybridized) linked via a honeycomb lattice with C grafted into the lattice. Thus, it is crucial to comprehend the compositional triangle of the BCN system while synthesizing controlled three‐phase‐N complexes to reduce the thermodynamically unstable segregation.^[^ ^43^ ^]^ As represented in **Figure** **2**, the triangular corners epitomize the pure phases. Graphene, in particular, and pure graphitic structures, in general, correspond to the pure phase at the C‐corners of the triangle. The B corner houses the recently prepared synthetic borophene, which is still being researched for optimizing its properties.^[^ ^58^ ^]^ Further, the transition from C to B corner creates a wide range of metastable intermediary phases, and the most stable ones are B_4_C_,_ B_2_C, and BC_3_.^[^ ^59^, ^60^ ^]^ It must be highlighted that the sp^3^ hybridization of the BC regions may interfere with the desired 2D layout of the BCN films. In continuation, the C–N face of the triangle lodges the layered C_3_N_4_ structures. Literature documents that graphitic‐C_3_N_4_ (g‐C_3_N_4_) is composed of covalent bonds between the atoms of C and N, wherein the first group of N atoms (N_1_) in each layer are coupled to three‐coordinated C atoms which are in the sp^2^ configuration and then a layer of g‐C_3_N_4_ is generated by the sp^2^ hybridization of C‐atoms with two‐coordinated N‐atoms (N_2_) into a triazine ring with 1,3,5. This configuration retains the two‐dimensionality of the film, presuming the establishment of C–N zones in the final structure. Furthermore, the bottom section of the triangle depicts the boron nitride nano morphologies. At the same time, the center refers to a 1:1 stoichiometry ratio of B: N. Thus, the BNs are comprised of sp^2^ configurations like the rhombohedral BN or sp^3^ hybridized structures like cubic (c‐BN), hexagonal‐BN (h‐BN), wurtzite BN (w‐BN), and diamond. Finally, the generation of BCN ternary compounds in the triangle is caused by the atomic resemblance of the B, C, and N atoms. Literature unveils that the combined characteristics of diamond and c‐BN produce c‐BCN with good hardness.^[^ ^61^, ^62^ ^]^ Also, the traits of 2D materials like graphene and h‐BN are combined to generate h‐BCN with tunable electrical features. 2D h‐BCN can be generated through well‐controlled (at BCN mid‐point) thickness and B, C, and N atomic ratios. However, it is challenging to maintain a precise atomic ratio during all conditions. Therefore, the final composition might deviate from the B:C:N‐1:1:1. The characteristics of ternary BCN‐based materials are particularly intriguing since they are an intermediary between the extensively researched carbon nitride and boron nitride.^[^ ^63^, ^64^, ^65^ ^]^

Understanding the crystal structures of graphene and h‐BN monolayers is crucial for comprehending the B–C–N structure. Graphene is a monolayer of C atoms bonded covalently in a honeycomb geometry. The AB sequence is observed in hexagonal graphitic structures, and weak van der Waals forces are suppressed during graphene extraction via graphite exfoliation. While in the h‐BN, they are out‐of‐plane van der Waals interactions and in‐plane s‐bonds with significant differences in the electronegativity between B and N, making them partially ionic s‐bonds. The fact that BN has a shorter interlayer distance than graphene is due to its semi‐polarity. Thus, in light of graphene and h‐BN structures, BCN is generalized to in‐plane and out‐of‐plane heterostructures, and the graphene layer can be packed in two h‐BN monolayers.^[^ ^43^, ^66^, ^67^, ^68^ ^]^

Saha and researchers have demonstrated that graphene/BN heterostructure was shown to have higher electrochemical efficiency than BN alone.^[^ ^69^ ^]^ They also proposed that the pseudocapacitive nature of the electrodes can be amplified primarily by the ionic nature of the boron nitride link in the BN structure and the change in the oxidation state of the BN bond during the electrolyte interaction. This effect of electrolytes on the electrochemical performance will be further discussed in Section 4.3.

**Figure 3:**
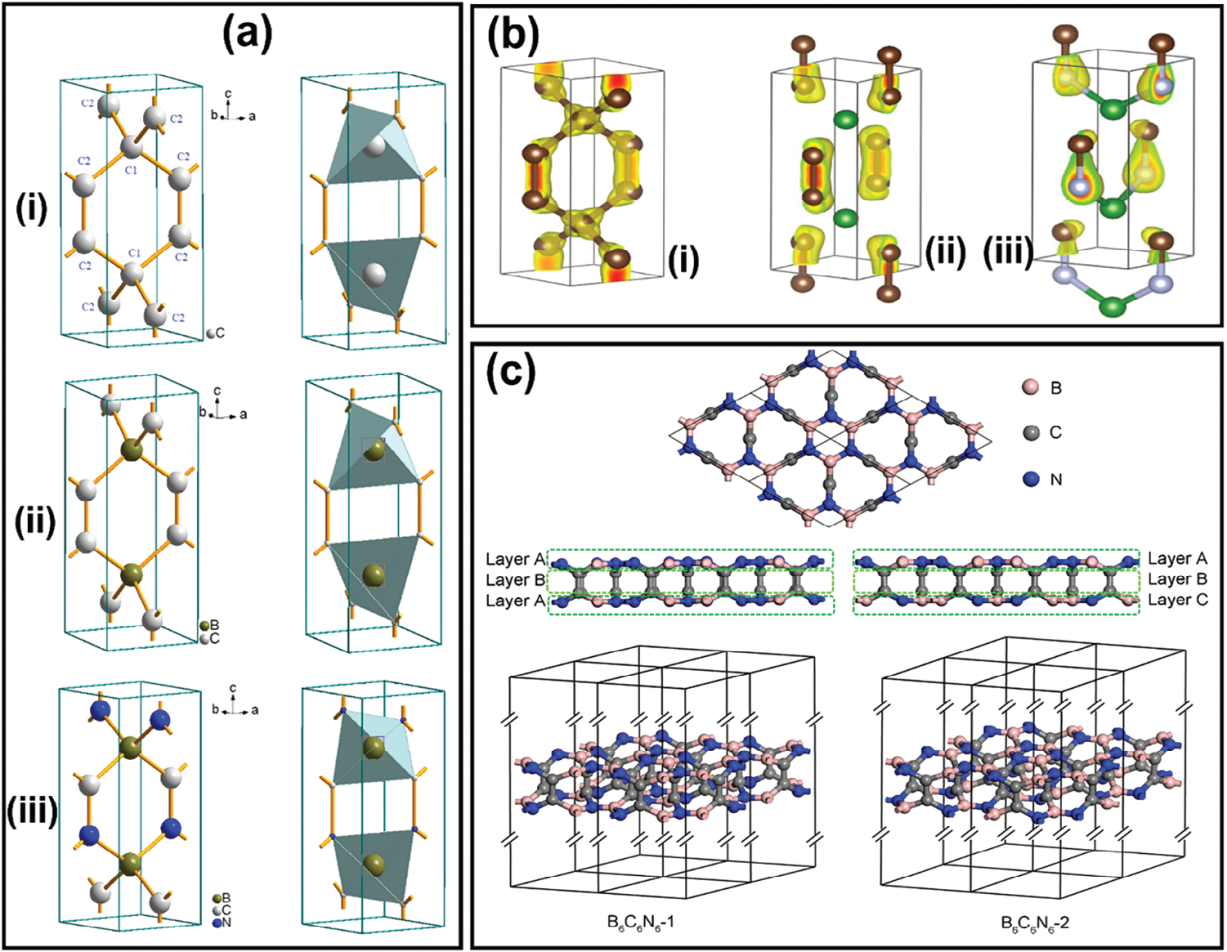
a) Tetragonal structure illustrations with projections of coordination polyhedral and b) Charge density projection (i) “glitter” C_6_, (ii) “glitter”‐based B_2_C_4_, and (iii) Equiatomic B_2_C_2_N_2_, (Reproduced with permission.^[^ ^70^ ^]^ 2023, Elsevier) c) ABA and ABC configurations designated as B_6_C_6_N_6_‐1 and B_6_C_6_N_6_‐2: Top and side perspectives of the BCN structures (Reproduced with permission.^[^ ^71^ ^]^ 2023, Elsevier).

Most recently, Mater and Solozhenko have proposed a novel equiatomic ternary BCN compound, which is structurally a “glitter” C_6_ composed of both trigonal (sp^2^ or C2) and tetrahedral (sp^3^ or C1) carbons^[^ ^70^ ^]^ (**Figure** **3**a(i)). The replacement of C1 by B prompts the hypothetic boron dicarbide (B_2_C_4_) (Figure 3a(ii)). This BCN crystal structure relates to the tetragonal space group P4_2_mc (Figure 3a(iii)) and is constructed by assembling corner‐sharing B_2_C_2_N_2_ tetrahedra. Their electron band structures unveil metallic traits, and the phase is energetically cohesive and stable with elastic properties (mechanical) and phonons band structurally (dynamically). The charge density projections depict the relationship between the crystal and electrical structures. The tetragonal phases C_6_, B_2_C_4_, B_2_C_2_N_2_, and the charge density yellow volumes are represented in Figure 3b. The replacement of C2 by N, which results in equiatomic B_2_C_2_N_2_, exhibits a differentiated charge envelope alongside the C–N pairs with the enormous yellow charge volume surrounding N (Figure 3b(iii)). In contrast, Lu and researchers have predicted two BCN structures through theoretical investigations, B_6_C_6_N_6_‐1 and B_6_C_6_N_6_‐2, which belonged to the P62m and P31m space groups. As illustrated in Figure 3c, both the BCN structures:^[^ ^71^ ^]^ i) have no imaginary frequencies hinting at their dynamic stability, ii) their elastic constants indicate the mechanical stability, and iii) ab initio molecular dynamics simulations demonstrated good thermal stability at RT and 1000 K.

Thus, the BCN system provides both mechanical and semiconducting applications. BN, a 2D hybrid material analogous to graphene, has recently been refurbished for electrochemical research.^[^ ^72^ ^]^ The heterostructure that results from the materialization of BN and carbonaceous material can enhance the electrochemical activity and charge carrier mobility.^[^ ^72^, ^73^, ^74^ ^]^

### 2.2 Electronic Traits

The electronic band structure of the BCN phase relies on two main factors: a) the varying atomic ratios of B_x_C_y_N_z_ and b) the distribution of atoms across the surface, which possibly leads to the formation of different types of bonds (C–C, B–C, N–C, B–N).^[^ ^75^ ^]^ However, the lack of practical information regarding the nanodomain distribution of graphene and h‐BN hinders the apprehension of the electronic structure of BCN materials. Furthermore, several studies have determined that the bandgap of BCN (>5.0 eV) can be finely monitored to magnify its catalytic activity by modifying the degree of C integration.^[^ ^76^, ^77^, ^78^ ^]^ The debate lies in whether h‐BN has an indirect bandgap transition with the valence band maximum (at K) and the conduction band minimum (at M) or if it has a direct bandgap (along K).^[^ ^75^ ^]^ In contrast, graphene is gapless and has two distinct Dirac points: K and K*’*.^[^ ^79^ ^]^ A complex band structure emerges by combining graphene and h‐BN in the BCN system, necessitating a thorough analysis using density functional theory (DFT). Furthermore, the bandgap of h‐BCN and the energy of the sub‐electronic states within the gap depend on the N and B atom ratios. Thus, increasing the number of B‐atoms introduces more holes, indicating the electron‐doped structure when N is dominant.^[^ ^80^ ^]^ The B_x_C_y_N_z_ exhibits magnified conductivity and higher carbon content. Foresee, 40% of carbon demonstrates ambipolar behavior, while at 90% carbon, an insulator‐to‐meta transition occurs in BCN. This transition may be attributed to graphene network percolation and hopping between BN edge states. Further, researchers Fellinger et al. have identified BC_3_N as a p‐type semiconductor for application as electrode material for the fields of electrocatalysis and electrochemistry.^[^ ^81^ ^]^ They report that BCN nanofilms exhibit carbon content‐dependent electrical resistivity. A comparative study between BCN and BC3N demonstrated that the specific resistivity decreases as the carbon content increases, potentially due to forming a percolation network involving carbon atoms.

**Figure 4:**
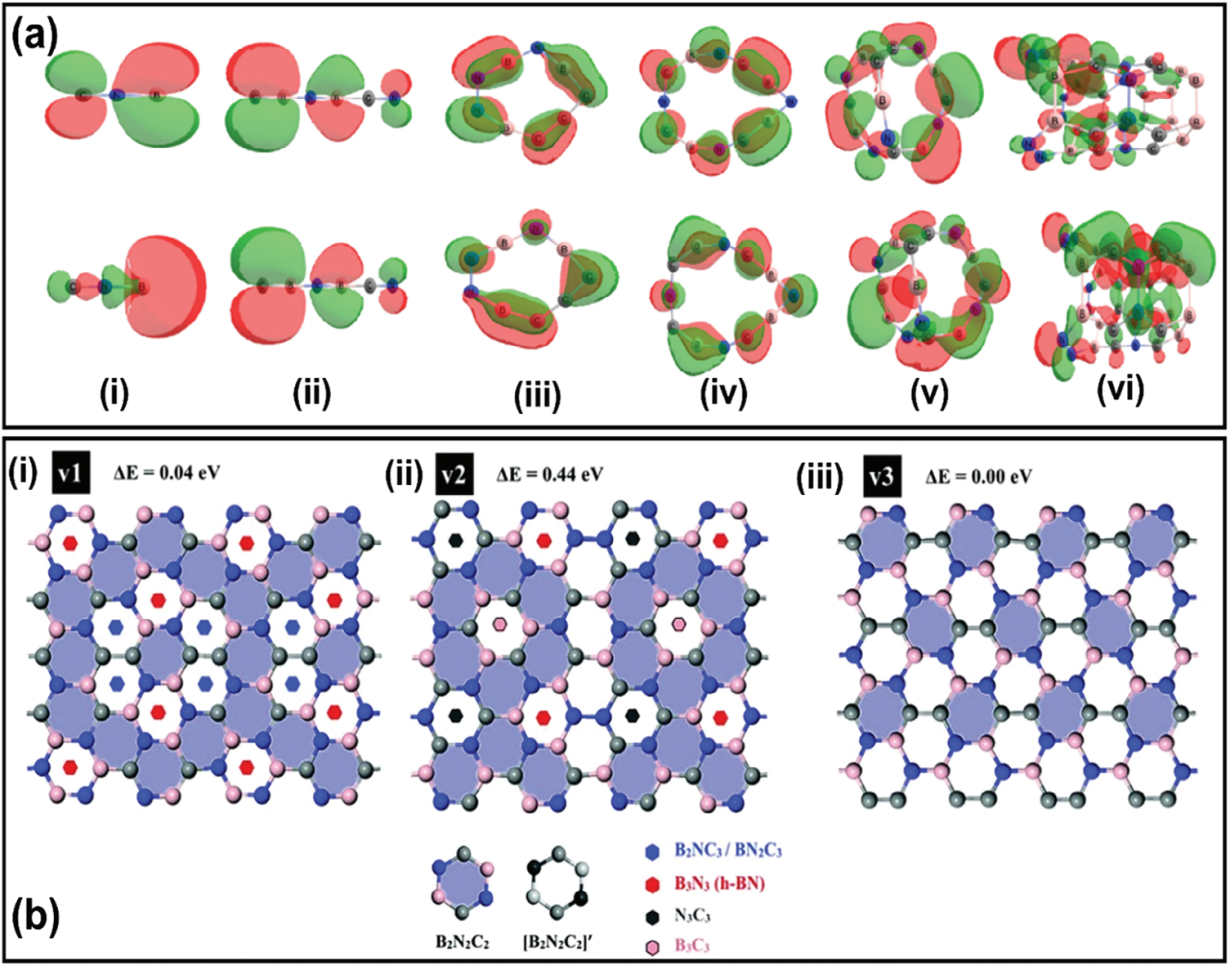
a) HOMO and LUMO frontier orbitals of BCN clusters (Reproduced with permission.^[^ ^82^ ^]^ 2023, Springer) b) Three possible monolayer structures of h‐BCN, namely (i) BCN(1), (ii) BCN(2), and (iii) BCN(3), (Reproduced with permission.^[^ ^84^ ^]^ 2023, RSC).

The latest study by Kumar et al. investigated the properties of (BCN)_x_ clusters (where *x* = 1–5), including (BCN)_12_ tubes employing first‐principle calculations.^[^ ^82^ ^]^ Their studies depicted that the cluster tended to become more stable as the number of BCN units increased, attributed to its incredible binding energy. Thus, the (BCN)_x_ clusters' relative stability differs according to the odd/even values of *x*. Furthermore, the electronic characteristics of these clusters were investigated via frontier orbital surfaces, their energies, the DOS curve, and several electronic parameters. The HOMO‐LUMO energy gap of (BCN)_x_ clusters and (BCN)_12_ tubes were both finite and similar to those of carbon nanotubes (**Figure** **4**a). This study may be of value while designing hybrid BCN‐based nanostructures. Fan and team have illustrated the electronic properties of P3m1‐BCN established on DFT investigations. The bandgap of indirect semiconductor P3m1‐BCN is 4.10 (4.06) eV, within generalized gradient approximation (GGA) at pressure zero.^[^ ^83^ ^]^ Additionally, the study reports the relationship between the bandgap and the pressure, and surprisingly, it depicted an enhancement in the bandgap with the increased pressure.

It is understood from these studies that the critical feature of BCN materials is their broad bandgap, which refers to the energy range that electrons are forbidden from occupying. This bandgap is pivotal to determining the materials' electrical conductivity for supercapacitor requisitions. BCNs typically have a wide bandgap of >5.0 eV based on the specific structure and composition. From structural composition, it is understood that BCNs can be tailored to exhibit desired electronic properties by modifying their compositional structure. Thus, doping BCNs with other elements can introduce impurity levels within the bandgap, allowing control over conductivity. Foresee, doping BCNs with carbon vacancies or carbon possibly generates n‐type and p‐type semiconductors, respectively.

### 2.3 Chemical Characteristics

#### 2.3.1 Oxidation Resistance

Although the oxidation resistance of BCN has yet to be thoroughly investigated, the data presented for h‐BN and graphene is used for its prediction. It has been claimed that h‐BN possesses an oxidation resistance of up to 1000 °C in air. Additionally, it can be thermally stable even at 1425 °C (under vacuum) and 3000 °C (inert gas flow conditions). At 400 °C, graphene begins to oxidize, and at 800 °C, there is total thermal dissociation. Therefore, compared to h‐BN and graphene, the BCN oxidation stability is more complex and hinges on the chemical composition.^[^ ^85^, ^86^, ^87^, ^88^ ^]^ Further, with oxygen on one side and B, N, and C on the other side, bonds may form. Fitzpatrick et al. investigated the effects of oxygen exposure on BCN film formed on germanium (Ge). They reported that single‐day exposure to an oxygen (O) environment causes 9 to 11% O. In contrast, two‐week exposure amplifies to just 13 to 15% O. Thus, according to their experimental findings, a carbon‐rich BCN with nearly the same thickness causes the structure to oxidize close to 18% O, whereas, N rich BCN is mainly prone to the surface oxidizing to more than 20% O. This susceptibility to oxidation diminishes dramatically as the BCN film becomes thicker.^[^ ^89^ ^]^

#### 2.3.2 Structural Stability

Researchers Deng et al. and Nehate et al. have demonstrated that higher C content resulted in less oxidation than N‐enriched films (CN_x_).^[^ ^15^, ^45^ ^]^ Thus, the overall chemical stability of the structure is governed by the relative energy of the structure resulting from various interatomic interactions between B, C, and N atoms. However, through their cohesive energy calculations, Thomas et al. report that not all BCN arrangements are thermodynamically stable (in terms of the relative energy of the structure).^[^ ^84^ ^]^ To exemplify these three structures referred to as BCN(1), BCN(2), and BCN(3) as modeled in Figure 4b wherein, the B_2_C_2_N_2_ stoichiometry is maintained despite having varied coordination environment surrounding the B, C, and N environments, however, with orientation and arrangement differences. Consequently, the B_2_C_2_N_2_ islands in each layer will be connected by various atomic bridges, giving rise to other hexagonal structures like BN_2_C_3_, B_2_NC_3_, B_3_N_3_, B_3_C_3,_ and N_3_C_3_. They also report that a structure (BCN(2)) with more B─C bonds is less stable because B─N and C─C bonds (BCN(3) and BCN(1)) are more robust than B‐C bonds. Thus, a structure is more stable when more C─C bonds are present. Therefore, among the three structures, the h‐BCN(3) model exhibits the most energetic stability.^[^ ^90^ ^]^

### 2.4 Mechanical Features

Hirata et al. demonstrated that BCN's hardness, Young's modulus, and stress values (≈285 GPa, 35 to 40 GPa, and 150 MPa) are more significant than h‐BN's (≈36.5 GPa, 1.5 to 1.3 GPa, and 1 to 16 MPa). They have exemplified that the carbon concentration affects the tribological characteristics of BCN.^[^ ^91^, ^92^ ^]^ With an increase in carbon, hardness rose from 11 to 18 GPa, which is attributed to sp^3^ C–N only despite the existence of sp^2^ C–C and sp^2^ C‐N. The presence of greater sp^2^ carbon domains served as self‐lubricants that caused the friction coefficient to decline from 0.4 to 0.2. Besides the inflation in B‐N content from 0 to 45%, the tensile fracture of the BCN layer during the B–N and graphene hybridization declined from 115 to 65 GPa. Researchers also suggested that boron and nitrogen affect the BCN film's hardness.^[^ ^93^ ^]^ Other studies, such as the simulations models of h‐BCN with three dissimilar configurations, exhibited a fracture strength of 81.4 to 93.4 GPa and fracture strain of 0.13 to 0.17 GPa, which was 35% and 21% lower than those of graphene.^[^ ^94^ ^]^ To complement this research, Fang et al. and Bohayra et al. have demonstrated a similar reduction with B, N doping (different atomic %) of graphene.^[^ ^75^, ^95^ ^]^ Foresee, if the temperature increases from 100 to 1000 K, the nuclear vibrations enhance, and the fracture strength diminishes. This trend is also seen along the armchair borders in h‐BCN.^[^ ^84^, ^92^ ^]^ Young's modulus also decreases with rising temperature, which could lead to plastic deformation. Thus, the strong covalent bonding between the B, C, and N atoms result in a robust lattice structure that can withstand high chemical reaction and temperatures. This stability is desirable for electronic devices requiring long‐term reliability and resistance to environmental factors. As mentioned, BCN's wide bandgap facilitates them as potential candidates for high‐temperature electronic device applications.

### 2.5 Optical Properties and Magnetic Qualities

BCN optical properties are derived from the h‐BN and graphene combination. Thus, the BCN bandgap can be tuned in between h‐BN and graphene; however, that depends on the composition. Nematollahi et al. and Yadav et al. have reported a bandgap of 2.6–5.5 eV for ternary BCN, demonstrating the scope for optoelectronic applications.^[^ ^96^, ^97^ ^]^ Further, BCN shows Raman‐active modes derived from graphene and h‐BN. In the ternary structure BCN, Raman modes are observed due to in‐plane atomic displacement or the *E*_2g_ phonon‐mode peak.^[^ ^98^, ^99^, ^100^ ^]^

The magnetic field‐induced electrochemical energy storage performance is an emerging possibility for supercapacitor research. The noncontact energy offered by the nanomaterial's magnetic field can influence a supercapacitor's electrochemical performance by generating molecular‐level changes at the electrode and electrolyte. High‐temperature ferromagnetism has garnered attention. BCN depicts ferromagnetism at RT. Studies have demonstrated^[^ ^101^, ^102^ ^]^ the occurrence of magnetic hysteresis at RT for graphene and h‐BN. Likewise, BCN materials have exhibited ferromagnetism at RT and are magnetically frustrated systems, as revealed by Kumar and researchers.^[^ ^50^ ^]^ BC_1.6_N prepared by a gas phase method depicted low magnetism. Sun et al. describe the ferromagnetism of BCN as a new candidate for new‐generation spintronic devices and optical field applications.^[^ ^103^ ^]^ Literature depicts several articles relating to this trait, which can be referred to as we prioritize properties that greatly favor supercapacitor performance in this review.^[^ ^99^, ^104^, ^105^, ^106^, ^107^, ^108^ ^]^ However, a comprehensive understanding of this Section is yet to be achieved with further research and exploration.

From the BCN triangular zone, it is evident that it has paved the way for new vistas for material research, which includes thin films and carbon nanostructures or materials prepared utilizing B, C, and N atoms. They can form the strongest covalent bonds, like those in pure carbon solids, leading to various unique properties for supercapacitor applications.

## 3 Methods of BCN Synthesis

Along with the properties, the electrochemical performance of BCN impinges on the variation of the distinguished dimensionalities, which can be synthesized through various bottom‐up approaches like chemical vapor deposition (CVD), physical vapor deposition, pyrolysis in the gas phase, solid‐state reaction method, molten salt method, wet chemical method, hydrothermal/solvothermal treatment, etc. The synthesis methods employed for BCN materials play a crucial role in tailoring their structural and electrochemical properties, directly impacting supercapacitor performance. By optimizing synthesis parameters and precursor selection, it is possible to achieve BCN materials with high specific surface area, excellent electrical conductivity, good charge storage capacity, and superior cycling stability, making them promising candidates for high‐performance supercapacitors. Each technique partakes in its unique way to contribute toward enhancing the supercapacitor performance comprised of BCN electrodes. As the synthesis strategies are not a prime focus of this review and could be found elsewhere,^[^ ^45^ ^]^ we have briefly discussed the advances in the following subsections given the supercapacitor performance of BCN side‐lining with it.

### 3.1 Chemical Vapor Deposition

**Figure 5:**
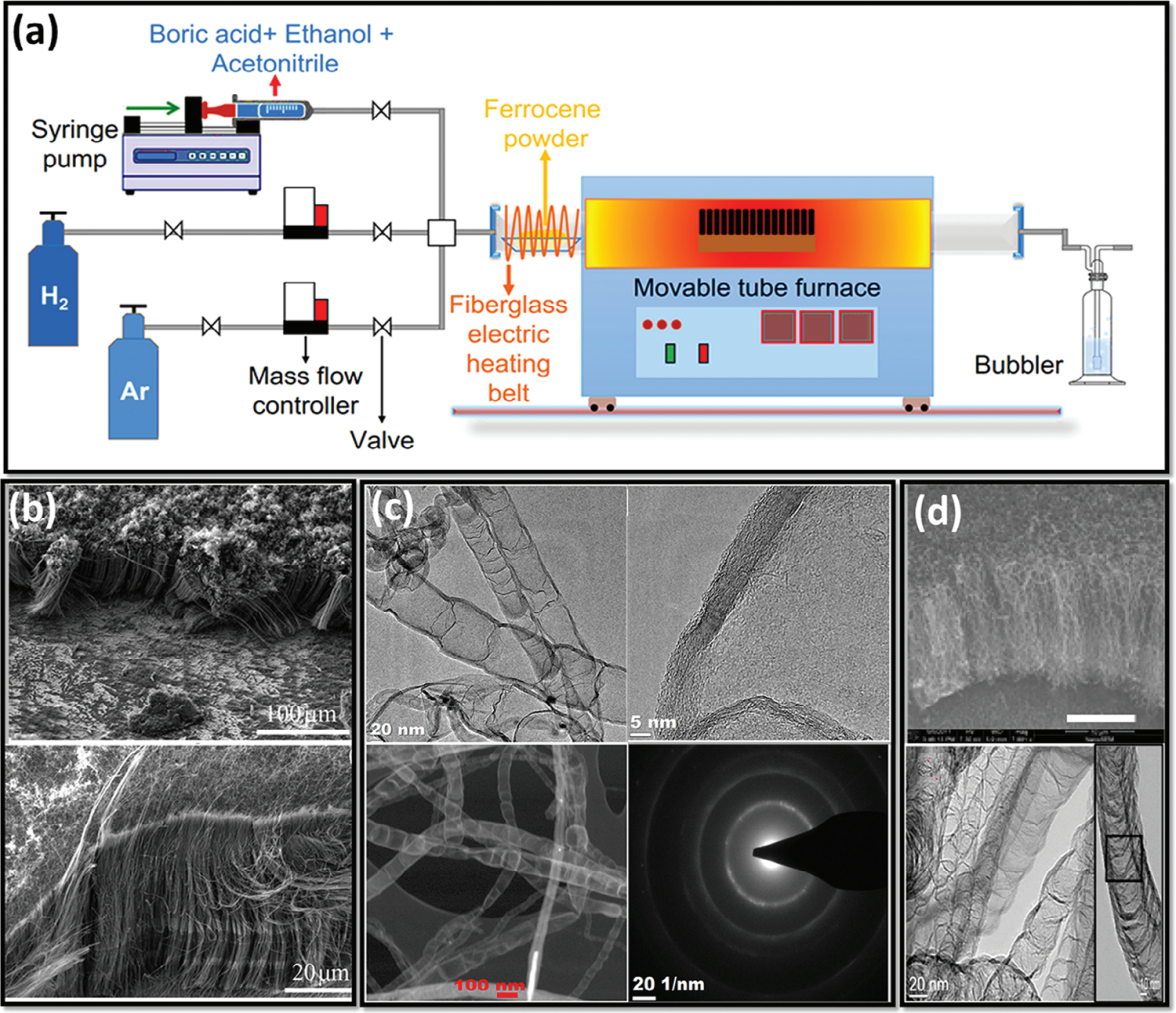
a) Schematic illustration of the synthesis protocol for BCN‐NTs. b,c) SEM and HRTEM images of BCN‐NTs, respectively (Reproduced with permission.^[^ ^110^ ^]^ 2023, Elsevier). d) SEM and HRTEM image of VA‐BCN‐NT arrays on SiO_2_/Si substrate (scale bar of 20 µm) (Reproduced with permission^[^ ^111^ ^]^ 2023, American Chemical Society).

Chemical vapor deposition (CVD) is a versatile technique for synthesizing 2D materials, including graphene and TMDs, offering scalability, high purity, and controllable growth parameters. Hence, fine‐tuning the reaction condition of the CVD process, which involves precursor selection, substrate choice, optimizing reaction growth conditions and parameters like temperature, gas flow, etc., utilizing specialized CVD equipment, and understanding the growth mechanism for successful synthesis of high‐quality BCN‐based supercapacitor electrodes. In the early 1970s, Kosalapova et al. synthesized B_x_C_y_N_z_ through the reaction of C and B in the presence of NH_3_/N_2_ at high temperatures.^[^ ^109^ ^]^ The concentration of the constituent atoms in BCN plays a pivotal role in its electrochemical performance, as discussed in Section 2. Given that, the starting material for synthesizing BCN is the primary deciding factor of its structural property. Hence, a lot of boron‐based precursors (BCl_3_, B_2_H_6_, trimethyl borazine, triethyl borazine, trimethylamine borane, triethylamine borane, tris‐(dimethylamino)borane, boric acid) and carbon‐based precursors (CCl_4_, hydrocarbons like C_2_H_2_, CH_4_, C_3_H_8_, acetonitrile, acetone, activated charcoal, activated carbon, graphite powder, graphene oxide, etc.) have been used to serve the purpose through plasma‐assisted CVD, microwave plasma enhanced CVD, graphene CVD, etc.^[^ ^45^ ^]^ Ertugrul et al. utilized floating catalyst chemical vapor deposition (FCCVD) to synthesize vertically aligned carbon‐rich BCN nanotubes (NTs) on a graphene substrate. A unique aspect of their approach involved incorporating an oxide layer analogous to CNTs, which served multiple functions.^[^ ^110^ ^]^ This oxide layer played a crucial role in promoting graphene growth, preventing the aggregation of catalyst nanodroplets, facilitating the formation of the desired graphitic carbon structure, and enabling the vertical growth of BCN‐NTs. The precursor materials, such as boric acid, ethylamine, and ethanol, provided B, N, and C sources, respectively. At the same time, the catalyst used was Bis(cyclopentadienyl) iron(II) at 950 °C with the introduction of argon (Ar) and hydrogen (H_2_) and utilizing a 1 wt.% boric acid solution in ethanol supplemented with acetonitrile for the 30‐minute growth process, followed by cooling to room temperature (RT). The detailed synthesis protocol is displayed in **Figure** **5**a. The obtained field emission scanning electron microscope (FESEM) and highresolution transmission electron microscope (HRTEM) images from their investigation are exhibited in Figure 5b, which suggested vertically aligned (VA)‐BCN‐NTs showed strong adhesion to graphene, forming a vertically oriented 3D composite structure characterized by high conductivity. This structure facilitated efficient charge transfer and provided a significant surface area. VA‐BCN‐NTs were densely grown multi‐walled structures measuring ≈60 µm in length, with diameters ranging from 40 to 80 nm. The introduction of B and N atoms into theCNT) structure induced notable changes in the microstructure of the nanotubes for higher stability of the electrode. BCN‐NTs displayed a stacked conical arrangement featuring bamboo‐like nanotubes with excellent crystallinity. The wave‐like structure observed on the BCN‐NTs walls and nanotubes' formation within the bamboo structure were attributed to B or N atoms within the graphitic carbon structure. This presence caused the rolling up of the graphite layer. The uniform atomic concentration of B and N atoms confirmed their homogeneous doping throughout the BCN‐NTs. Selected area electron‐diffraction (SAED) confirms the presence of the graphitic structure from the formation of the rings (Figure 5c).^[^ ^110^ ^]^

Likewise, Dia and his co‐workers synthesized VA‐BCN‐NT arrays in SiO_2_/Si substrate having the starting material as melamine diborate (pre‐synthesized by the reaction of melamine and boric acid), and the temperature was kept at 1000 °C. The SEM images clearly stated the formation of vertically aligned BCN‐NTs, which was again validated through the TEM images, implying the formation of 25–30 nm of diameter and 10 nm of the wall thickness of BCN‐NTs (Figure 5d). However, the surface area for this group (347 m^2^ g^−1^) was relatively high compared to the above group (97 m^2^ g^−1^), so the supercapacitive performance can be attributed to the reaction conditions, substrate, and the precursors. Similarly, loads of other reports are available that preview the synthesis of BCN, which might be helpful for many researchers in this field. Nevertheless, fine adjustment in reaction conditions (optimum temperature below 1000 °C), precursors (mainly inorganic ones), and choosing appropriate substrates can be crucial to fabricating BCN‐based porous and stable supercapacitor electrodes through the CVD technique.

### 3.2 Pyrolysis

**Figure 6:**
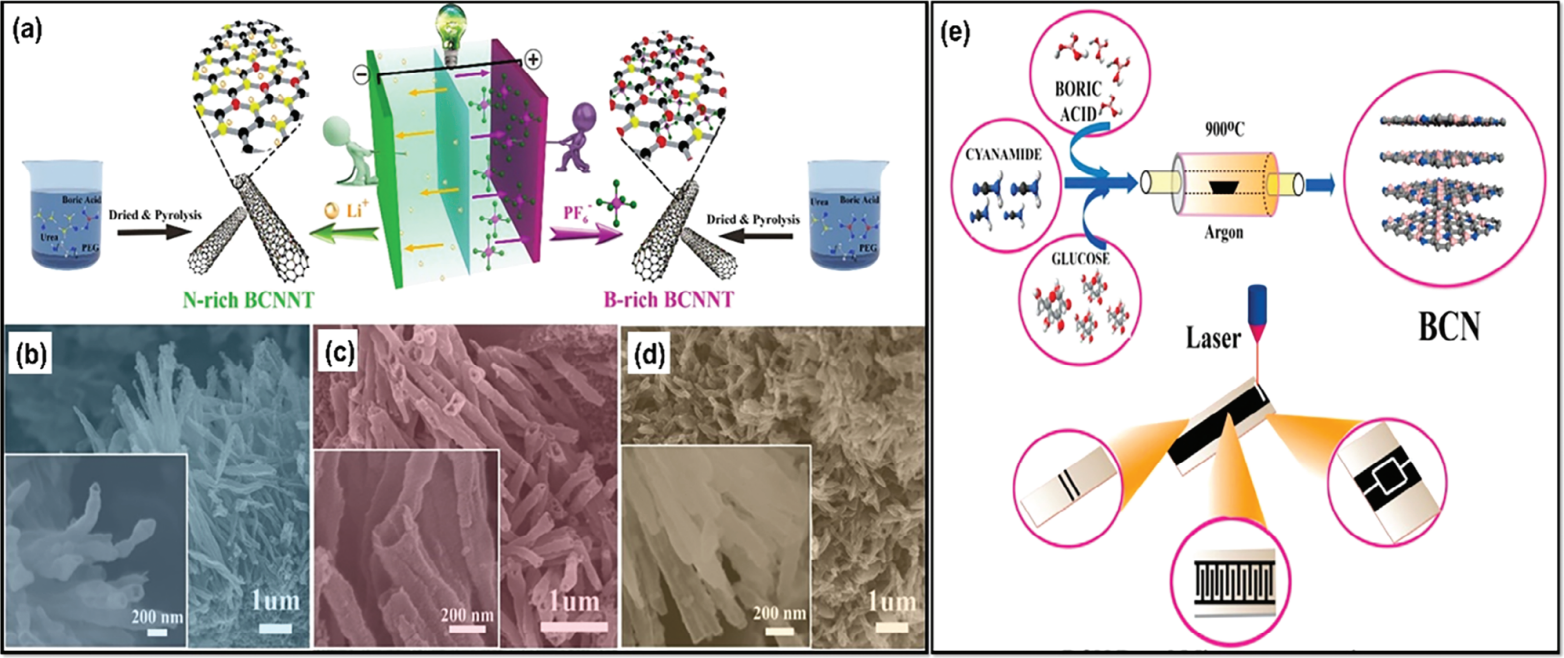
a) Schematic representation of different BCN nanotubes synthesis, b–d) SEM images of BCN nanotubes, N‐rich BCN, and B‐rich BCN nanotubes, (Reproduced with permission^[^ ^112^ ^]^ 2023, American Chemical Scoiety). e) Synthesis protocol of BCN nanosheets through different starting materials (Reproduced with permission^[^ ^113^ ^]^ 2023, Elsevier).

The pyrolysis method is risk‐free compared to other synthesis techniques like CVD, physical vapor deposition (PVD), or solid‐state reaction methods. It involves fewer controlling parameters, allowing it to fabricate BCN electrodes on a large scale with excellent thermal stability, high hardness, and potential applications in energy storage. In general, pyrolysis involves heating of the slurry made up of respective precursors of BCN; Jiang and their co‐workers came up with an innovative strategy for a wide potential window of the lithium‐ion capacitor by using electropositive boron‐rich BCN and electronegative nitrogen rich BCN nanotubes. By considering a suitable directing agent, polyethylene glycol, they varied the concentration of boric acid and urea to fabricate both the BCN as mentioned above species, which aided in high electrochemical active sites, defect‐rich electrode material along with sufficient energy for lithium‐ion adsorption leading to higher capacitive performance.^[^ ^112^ ^]^ The synthesis method and SEM images of BCN, N‐rich‐BCN, and B‐rich BCN are displayed in **Figure** **6**a–d. Karbhal et al. successfully synthesized BCN sheets by dissolving equimolar ratios of boric acid, glucose, and cyanamide in water. Subsequently, they heated them at 70 °C until they transformed into a dense paste and thoroughly dried. The resulting dried material was placed in a ceramic boat, which was subjected to a temperature of 900 °C for a duration of 3 h within an Argon atmosphere using a tubular furnace (Figure 6e). This work fabricated a flexible yet strong graphene/nickel‐based composite paper (RGN paper) with a one‐step cold compression method at RT. The cellular structure of nickel foam allows combining metal nickel and graphene sheets to construct sandwich‐structured composite films with flexibility and robustness. The RGN paper shows not only exceptional electromagnetic interface (EMI) shielding effectiveness (55 and 85 dB in the X‐band while the thickness is merely 0.12 or 0.25 mm) but also exhibits high in‐plane thermal conductivity (247 W (m^−1^ K^−1^), much higher than pure nickel). The ultrahigh EMI shielding performance is attributed to the high electrical conductivity of the RGN paper and the unique built‐in nickel network. In addition, the RGN paper also indicates high flexibility and mechanical robustness after repeatedly bending at a strain of 80% for 1000 cycles.^[^ ^113^ ^]^ Later, the synthesized BCN was laser patterned to fabricate a micro supercapacitor successfully. Likewise, Sreeraj and the group synthesized carbon‐rich BCN by following a similar approach by altering the starting materials as boric acid, activated charcoal, and urea. In both studies mentioned above, the synthesized BCN showed a 2D sheet‐like morphology with ample electrochemical active edges, which was fruitful in their supercapacitor performance.^[^ ^36^ ^]^ However, the electrochemical performance of Zou et al. synthesized BCN 3D network outranked the above studies by replacing the precursors with melamine foam‐derived carbon skeleton, providing higher surface area and as a reservoir for electrolytic ion intake. However, the solvent‐free BCN network was synthesized through two‐step methods: ball milling of B_2_O_3_, dicyandiamide, and polyvinylpyrrolidone and afterward transferring it for pyrolysis at 1050 °C in the presence of H_2_/N_2_. Their study showed the highest interlayer spacing of 0.38 nm and high porosity compared to the abovementioned investigations. Hao et al. prepared porous BCN with a wide bandgap through freeze‐drying of urea, boric acid, and glucose in an N_2_ atmosphere.^[^ ^114^ ^]^ Similarly, other synthesis reports inspected a similar pyrolysis method for synthesizing BCN electrodes for supercapacitors by varying the starting materials and reaction conditions.^[^ ^115^, ^116^, ^117^ ^]^

### 3.3 Hydrothermal/Solvothermal Treatment

**Figure 7:**
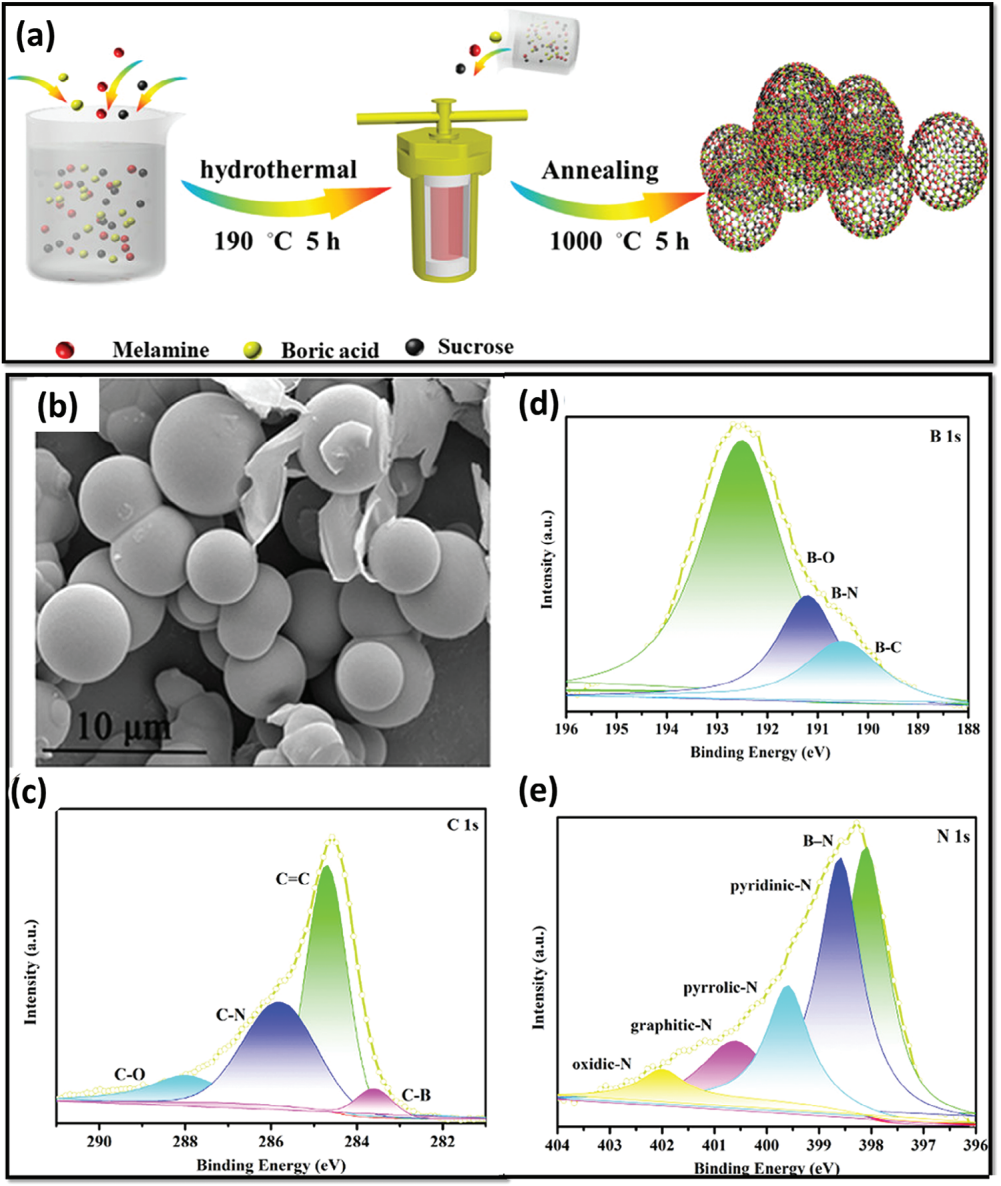
a) Pictorial representation of the synthesis procedure, b) SEM image of BCN micropores, c–e) deconvoluted peaks of B 1s, C 1s, and N 1s along with their polar bonds. (Reproduced with permission.^[^ ^119^ ^]^ 2023, American Chemical Society).

CVD and pyrolysis techniques need high temperatures and many reaction conditions to optimize the electrode material. However, hydrothermal/solvothermal treatment undergoes a low‐temperature route and provides high purity, crystallinity, controlled morphology, and size of the BCN electrodes. It not only aids in the high yield of samples but is also responsible for tunable chemical and physical properties of the samples. Xu et al. synthesized vertically‐aligned BCN nanotubes (VABCNNTs) through a two‐step facile solvothermal method in which sodium azide, ammonium fluoroborate, and hexadecyl trimethyl ammonium bromide were taken in a certain amount and dissolved in pre‐distilled methyl cyanide and benzene. Lastly, the solution was transferred to an autoclave and kept at 400 °C for 14 h in a furnace. As usual, the final product was obtained after washing the cooled‐down solution with water, ethanol, and HCl. BCN exhibited a cleverly designed configuration characterized by vertically oriented morphology that lacks transverse layers, resulting in an exceptionally elevated capacitance. Their study showed that the polar bond structure, porous unbuckled pore channels, and high surface area attributed to the increased intake of electrolytic ions, thus, high capacitance.^[^ ^118^ ^]^ Yang and colleagues successfully showcased a scalable and cost‐effective technique for fabricating BCN's 3D microspheres.^[^ ^119^ ^]^ The fabrication process involved a hydrothermal treatment followed by annealing. By employing a stoichiometric ratio of melamine, boric acid, and sucrose, the researchers conducted the hydrothermal treatment at 190 °C for 5 h, resulting in microspheres with enhanced surface area, electrical conductivity, and abundant functional groups. Subsequently, carbonization was carried out at 1000 °C for 5 h to create highly porous 3D BCN micropores, enabling a high‐rate capability. The schematic representation of the synthesis procedure is given in **Figure** **7**a. The SEM images of the 3D micropores are presented in Figure 7b, while X‐ray photoelectron spectroscopy (XPS) measurements are deconvoluted and exhibited in Figure 7c–e. The presence of the functional groups in BCN offers advantages in the energy storage process, which is observed from the Fourier‐transform infrared spectroscopy (FTIR) and XPS. The pyrrolic‐N‐functional group exhibited favorable interaction with electrolyte ions, while the quaternary N‐functional group significantly enhanced the electronic conductivity of the 3D‐BCN‐4 microspheres.^[^ ^119^ ^]^ Another exciting report showed the green synthesis of BCN sheets through the solvothermal method by using boric acid 2,4,6‐Tri(2‐pyridyl)−1,3,5‐triazine in ethylene glycol at 250 °C for 24 h followed by annealing at 400 °C for 2 h. in the presence of N_2_. The 2D BCN sheets exhibited high capacitance owing to their ample electrochemical active edges and high surface area. Overall, hydrothermal/solvothermal, compiled with annealing, can be a considerable technique for synthesizing BCN. This can be ascribed to its time effectiveness, large‐scale production, high crystallinity, large surface area, and plentiful active sites.^[^ ^120^ ^]^

### 3.4 Other Methodologies

**Figure 8:**
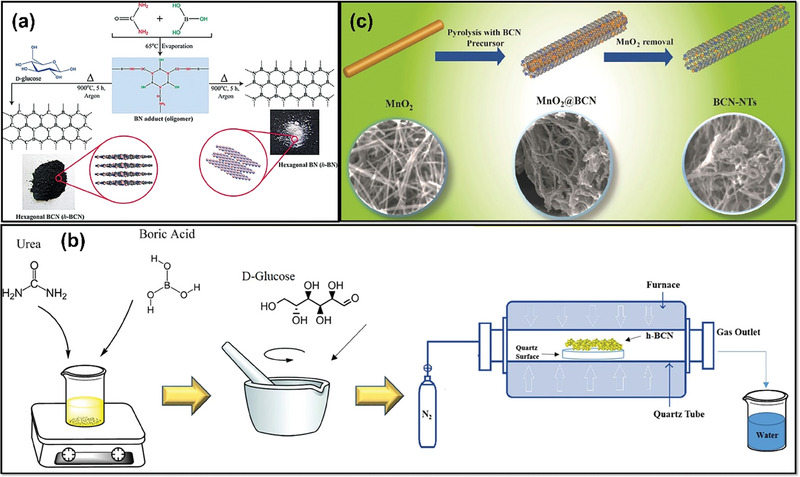
a) Schematic illustration of the synthesis of BCN bulk sheets (Reproduced with permission.^[^ ^123^ ^]^ 2023, Wiley). b) Three‐step template free synthesis pictorial representation of BCN sheets (Reproduced with permission.^[^ ^124^ ^]^ 2023, Elsevier). c) Pictorial representation of the template‐assisted synthesis of BCN (Reproduced with permission.^[^ ^126^ ^]^ 2023, Wiley).

Apart from CVD, pyrolysis, and hydrothermal methods, there are some other synthesis techniques, such as the wet chemical method,^[^ ^121^ ^]^ the molten salt method,^[^ ^122^ ^]^ the template‐assisted method,^[^ ^122^ ^]^ etc. However, these synthesis methods can be categorized together in this section owing to their applicability and usage. Karbhal et al. synthesized 2D BCN sheets using urea and boric acid as boron and nitrogen precursors in the wet chemical method, while glucose served as the carbon source. The synthesis involved preparing a BN adduct from urea and boric acid and mixing it with glucose in different weight ratios. Heating the mixture at 900 °C yielded BCN nanosheets. For comparison, separate heating of glucose and BN adduct produced carbon and h‐BN, respectively.^[^ ^123^ ^]^ **Figure** **8**a shows the schematic diagram of the synthesis procedure. The collected product was highly crystalline and possessed a high surface area. Similarly, Mirzaee et al. synthesized BCN with an increased surface area by utilizing boric acid and urea in a molar ratio of 1:48. The mixture was slightly heated to 65 °C, resulting in the formation of white needle‐like structures. This product was then combined with glucose in a 1:1 molar ratio and ground in an agitate mortar for 5 h. Subsequently, it was subjected to a temperature of 900 °C under an N_2_ atmosphere for 1 h (Figure 8b). Analysis of FESEM images revealed a 2D sheet‐like architecture for the prepared BCN, ranging in size from 0.3 to 2.0 µm. The nanosheets displayed a stacked and crumpled morphology with twisting patterns.^[^ ^124^ ^]^ In the case of the template‐assisted method,^[^ ^125^ ^]^ to achieve a reduced synthesis temperature and incorporate specific structural characteristics into the prepared BCN, a 3D mesoporous carbon nanocage template was utilized (Figure 8c). This template possessed a large surface area and a porous architecture in cages. Boric acid was employed as the boron precursor, and the template was placed on a graphitic crucible. The reaction is maintained at 1350 °C for 45 min. In the presence of nitrogen gas.^[^ ^125^ ^]^ The resulting BCN unveiled a uniformly oriented structure with interconnected mesopores in a 3D configuration covering the product uniformly. Other methods that involve synthesis and disposal and require neutral experimental conditions tend to be time‐consuming and have limitations due to the sensitivity of the precursor to air. Hence, template‐free methods are often preferred for large‐scale production of BCN due to their reduced number of synthesis steps and precursor consumption. Furthermore, **Table** **1** provides the merits and demerits of the various methods employed for BCN synthesis.

**Table 1:** Merits and demerits of the various methods employed for BCN synthesis.

Synthesis Method	Advantages	Disadvantages
CVD	Versatile and scalable.	Requires high‐temperature conditions.
	High purity and controllable growth.	Precursor selection complexity.
	High‐quality BCN materials possible.	Resource‐intensive.
	Specialized CVD equipment for control.	
Pyrolysis	Lower risk and fewer controlling parameters.	Requires optimization of precursor selection.
	Suitable for large‐scale production.	May not achieve high surface area like CVD.
	Tuning electrochemical properties via precursors.	Limited control over growth conditions.
	Minimal resource requirements.	
Hydrothermal/Solvothermal Treatment	Operates at lower temperatures.	Limited to specific precursor combinations.
	High purity, crystallinity, and controlled morphology.	Requires precise control over the reaction.
	High yield and tunable chemical/physical properties.	Sensitive to solvent/precursor selection.
	Abundant active sites.	Scalability may vary.
Other Methods	Offers diverse techniques.	Vary significantly in complexity and resources.
	Potential for unique structural characteristics.	Precursor sensitivity and control requirements.
	Suitable for specific research/production needs.	Some methods may involve time‐consuming steps.
	May require specialized templates or conditions.	Scalability may be limited.

## 4 Factors Affecting the Electrochemical Performance of BCN

Several factors impact the performance of BCN electrodes in energy storage systems. To begin with, the BCN material's composition and structural properties considerably influence its conductivity, surface area, and stability. Based on the stochiometric ratio of B, C, and N, a variety of hybridized and randomly distributed graphene and h‐BN domains B_x_C_y_N_z_ system (0 ≤ *x*, *y*, *z* ≤ 1) with constitution ranging from pure‐ h‐BN to pristine graphene can be formed. Pure h‐BN is an insulator, while pure graphene is a material with a zero‐bandgap. Because graphene and h‐BN have comparable honeycomb structures, h‐BCN material inherits h‐BN's outstanding chemical and thermal stabilities. It has a narrower bandgap and better electrical conductivity due to carbon doping. The optical, mechanical, magnetic, and electrical properties of B_x_C_y_N_z_ materials can be enhanced by modifying the arrangement and atomic ratios of B, C, and N atoms. BCN semiconductors, a hybrid phase of h‐BN and graphene, have an adjustable bandgap and higher charge carrier mobility than isolated h‐BN. Simultaneously, doping h‐BN domains offers BCN more oxidation resistance than graphene. BCN has enormous potential in supercapacitor applications due to its unique planar structure, excellent electrical and thermal conductivity, large specific surface area (SSA), and readily regulated components and thicknesses.^[^ ^127^ ^]^ Second, the shape and architecture of the electrode, such as thickness, porosity, and surface roughness, influence ion diffusion and charge storage capacity. Furthermore, the electrolyte and its compatibility with BCN electrodes are critical in determining its overall performance. The following key factor affecting BCN's performance is the interlayer spacing. The interlayer distance of graphite and h‐BN is too small to accommodate ions such as sodium (Na^+^). Modifying B and N helps enhance the interlayer distance.^[^ ^126^ ^]^ The BCN (002) plane shows disordered nanosized domains with interlayer diffraction. As the size of the graphene domain rises, the (002) plane 2θ progressively shifts from 26.9° to 24.5° while the peak broadens and the intensity decreases. A rise in interlayer spacing causes a change in peak position, but the intensity variation indicates defects.^[^ ^43^ ^]^ The studies were done by Lei et al.^[^ ^128^ ^]^ and Wang et al.^[^ ^114^ ^]^ also demonstrate that interlayer spacing increases with increasing the proportion of carbon. Using XRD, Gao et al. found the interlayer spacings between two adjacent layers of h‐BN, graphene, and G/h‐BN nanosheets to be 3.334, 3.383, and 3.356 Å, respectively.^[^ ^129^ ^]^ The studies done by Yu et al. demonstrate that the d‐spacing was found to decrease and crystallinity improved with boron concentration.^[^ ^122^, ^130^ ^]^ In addition to the proportion of B and C, doping was also found to increase the interlayer spacing of BCN. The fluorine doping helps to enhance the interlayer spacing to 3.89 Å, generating many electrochemical active sites and promoting ion diffusion.^[^ ^131^ ^]^ Similarly, the heterostructure formation using MXene^[^ ^132^ ^]^ increases the interlayer spacing to 24.9^o^ of BCN, enhancing the electrochemical activities. It is also necessary to discuss the temperature dependence of the BCN diffraction patterns. Elevating the temperature has been shown to diminish the full width of the half maximum (FWHM) of the (002) and (100) peaks, indicating an increase in crystallite/grain size.^[^ ^133^, ^134^ ^]^ Finally, electrode–electrolyte interface, charge transfer kinetics, and cycle stability all impact the efficiency and lifetime of BCN‐based electrodes in microsupercapacitors. Understanding and managing these parameters is critical for increasing the performance of BCN electrodes and the devices' overall energy storage capacity. Through the considerations mentioned above, the next part vividly studied the parameters influencing the charge storage performance of the BCN electrode.

### 4.1 Influence of BCN Morphology on the Energy Storage

BCN, which has a similar shape to graphene, has demonstrated outstanding energy storage properties as a negative electrode due to increased charge density and electronic spin. The nitrogen and boron atoms in the carbon framework in BCN have varying electronegativities that will break the carbon's electroneutrality and improve discharge/charge capability and electrical conductivity.^[^ ^119^ ^]^ The morphology of BCN contributes to its electrochemical performances to an extent. For example, the 2D structures of BCN provide more significant electrical contact for charge transfer by interlocking between individual units and enhancing topographical overlapping. Several literature studies demonstrated the benefits of 2D BCN in supercapacitor applications. For example, Kumar et al. synthesized a few‐layered 2D BCN structure using a carbodiimide reaction that created amide bonds between the amine groups on the BN sheets and the carboxylate groups on the graphene sheets, aids in the layer‐by‐layer self‐assembly of BCN.^[^ ^135^ ^]^ This layered material demonstrates satisfactory performance as an electrode in supercapacitors. However, these traditional BCN nanosheets, similar to other 2D materials, face similar difficulties, such as self‐restacking. To overcome this problem, Tu et al. modified BCN's morphology by controlling the carbon content from conventional nanosheets into 3D BCN microspheres. These 3D structures effectively reduce self‐restacking and also provide additional electrochemically active sites. Furthermore, 3D‐BCN microspheres have a significantly higher specific surface area than BCN 2D nanosheets, allowing for more space to expedite ion transport and enhance the number of active sites.^[^ ^119^ ^]^ Another morphology called Ravine‐like BCN was reported by Chen et al. for supercapacitor applications. This morphology helps to increase the contact area between the electrolyte and electrode and facilitates easier ion exchange. This BCN‐based supercapacitor shows a specific capacitance of nearly 805.9 F g^−1^ in 0.2 A g^−1^ with excellent stability of ≈91%.^[^ ^117^ ^]^ 1D BCN also performed very well as negative electrode materials for supercapacitor applications. This can be due to their excellent electrical conductivity and structural advantages, guaranteeing enough ion diffusion channels during electrochemical charging and discharging. Tabassum et al. used a synthetic method using urea, boric acid, and polyethylene glycol (PEG) to produce uniformly dispersed BCN nanotubes on the melamine formamide (MF)‐derived 3D carbon skeleton was used. This interconnected BCN nanotube over 3D carbon architecture shows excellent potential for use in electrochemical energy storage applications. Both symmetric and asymmetric devices exhibited excellent supercapacitor performances with energy densities as high as 19.8 and 72 Wh kg^−1^.^[^ ^136^ ^]^ **Table** **2** summarizes the effect of BCN morphology on the supercapacitor performances.

**Table 2:** Effect of morphology on the supercapacitor performances.

Electrode	Precursor and synthesis method	Morphology	Specific capacitance	Capacitance retention and coulombic efficiency	Ref.
BN_1–x_G_x_ composite	Graphene with boric acid and urea – Carbodiimide reaction	2D sheets	238 F g^−1^ at 0.3 A g^−1^	98% after 1000 cycles	[135]
BCN microspheres	Sucrose, boric acid, and melamine – Hydrothermal followed by carbonization	3D microspheres	899 F g^−1^ at 1 A g^−1^	94% after 10 000 cycles	[119]
BCN	2,4,6‐tri(2‐pyridyl)−1,3,5‐triazine and boric acid – Refluxing followed by carbonization	Ravine‐like	805.9 F g^−1^ at 0.2 A g^−1^.	91% after 3000 cycles	[117]
Asymmetric device 3BCN‐950//65 Ni‐MDH	Polyethylene glycol (PEG‐2000), urea, and boric acid ‐Carbonization	Nanotubes	344 F g^−1^ at a current density of 1 A g^−1^	80.7% after 10 000 cycles	[136]

**Figure 9:**
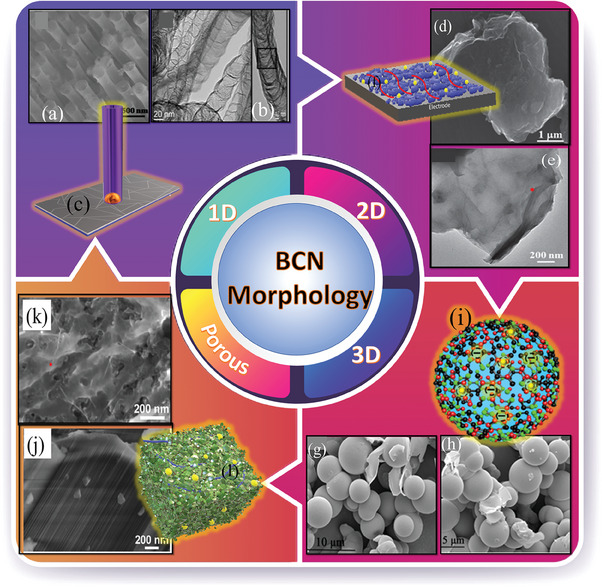
Various types of morphology of BCN. a,b) 1D structures of BCN (Reproduced with permission from Scientific Reports 4, 2014, 6083 and reproduced with permission.^[^ ^111^, ^118^ ^]^ 2023, Americal Chemical Society). c) Ion transfer pathway in 1D structures, d,e) 2D BCN nanosheets. (Reproduced with permission.^[^ ^135^ ^]^ 2023, John Wiley and Sons). f) Ion transfer pathway in 2D structures g,h) 3D Structures of BCN, i) Ion transfer pathway in 3D structures. (Reproduced with permission from ACS Applied Material Interfaces 12, 2020, 47416, American Chemical Society).^[^ ^119^ ^]^ j,k) BCN porous structures, l) Ion transfer pathway in BCN porous structures, (Reproduced with permission.^[^ ^114^ ^]^ 2023, American Chemical Society).

Thus, the inherent porous structure of BCN microspheres allows effective electrolyte penetration, shortening the electrolyte ion transport and diffusion channel lengths and providing high charge–discharge rates and fast kinetics. As a result, rational modification of the morphologies of BCN electrodes (**Figure** **9**) for the requirements of high‐performing energy storage systems is critical to obtain increased performance.

### 4.2 Electrode Architecture

**Figure 10:**
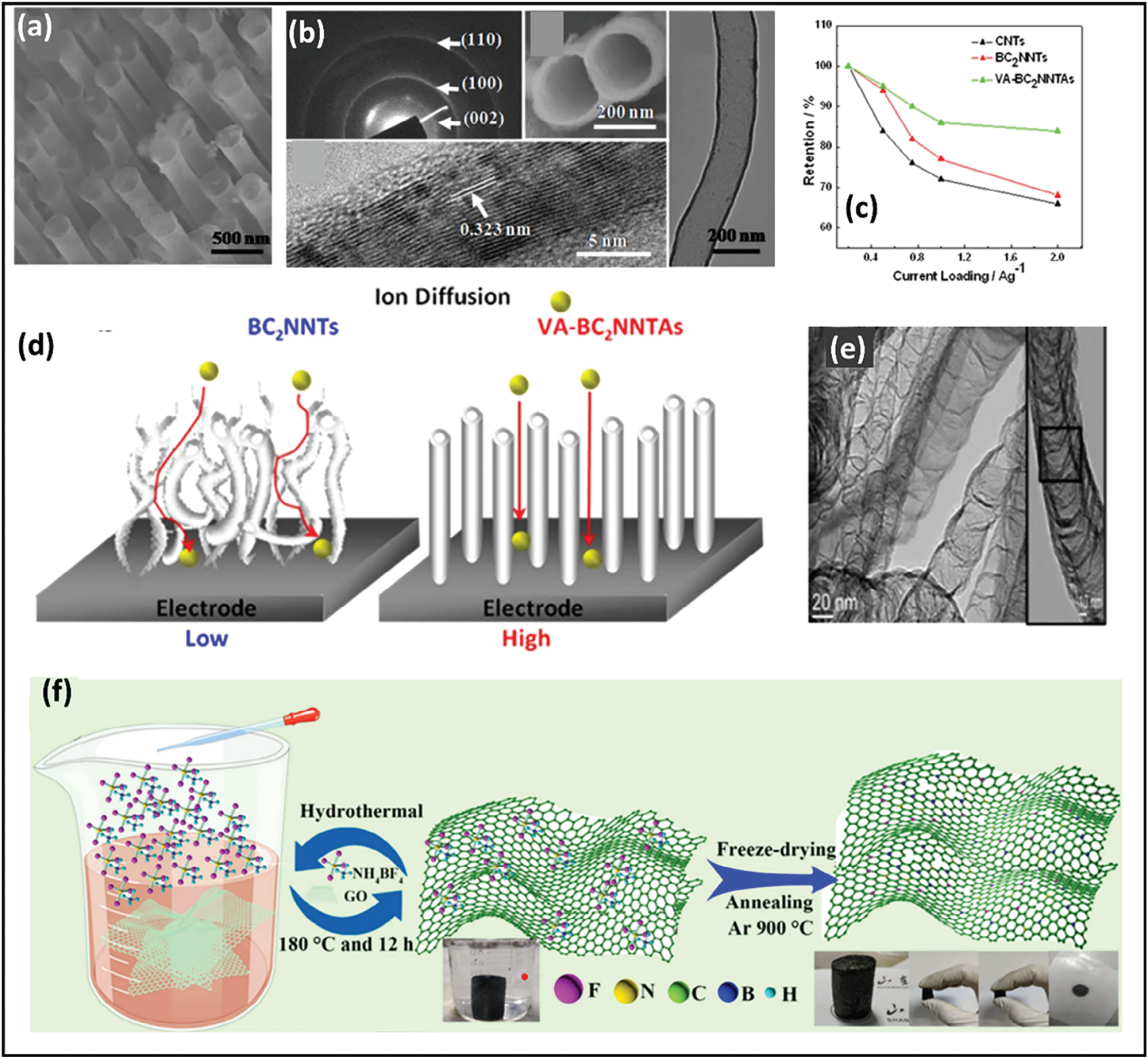
a) FESEM images of VABCNNTs, b) Lower and higher magnification TEM images of VABCNNTs, c) Specific capacitance graph showing enhanced performance of VABCNNTs, d) Schematic diagram showing ion diffusion for both BCNNTs and VABCNNTs, (Reproduced with permission.^[^ ^118^ ^]^ 2023, Scientific Reports). e) TEM images of VA‐BCNs (Reproduced with permission.^[^ ^111^ ^]^ 2023, American Chemical Society). f) Schematic diagram showing the preparation of F‐BCN aerogel (Reproduced with permission.^[^ ^131^ ^]^ 2023, John Wiley and Sons).

Optimization of the electrode architecture is also crucial in increasing capacity. Nanostructured electrodes such as nanowires, nanosheets, and nanoparticles have been developed to enhance the electrochemical performance of supercapacitors. In practice, these nanostructured electrodes frequently include electrode preparation methods, which unavoidably result in several problems. Using organic binders to prepare electrodes buries active areas and restricts mass transfer, potentially underutilizing electrochemical performance benefits. Furthermore, due to the low conductivity of nanostructured electrodes, a high concentration of conducting agents is required, which is easily etched at higher potential. Thus, vertically aligned BCN nanotube arrays (VA‐BCNNTAs) are an excellent choice as a conductive scaffold in supercapacitor applications. Zhou et al. reported a supercapacitor electrode material based on VA‐BCNNTAs synthesized using the solvothermal method for the first time. The aligned shape of VA‐BCNNTAs (**Figure** **10**a,b) may efficiently allow electrolyte ion transportation, and the non‐buckled tubular construction ensures a high surface area to ensure plentiful electrolyte ion transport throughout the charge/discharge process. The polar bond nature of VA‐BCNNTAs and the aligned unbuckling hollow tube construction may contribute to their high specific capacitance. When compared to vertically‐aligned carbon nanotubes (VA‐CNTs) having nonpolar bonds and nonaligned BCN nanotubes (BCNNTs), VA‐BCNNTAs have the most significant specific capacitance, outstanding rate capability, and high durability, making them appealing as electrode materials for supercapacitor applications. In electrochemical studies, VA‐BCNNTAs exhibit a more extensive CV with a rectangular‐like shape, indicating a thicker double‐layer development due to heteropolar B‐N bonding, which results in an extra‐dipole moment. It should be noted that the electrochemical performance of randomly entangled BCNNTs electrodes exhibits a relatively low capacitance (70.18 F g^−1^) when compared to VA‐BCNNTAs (547 F g^−1^) in 6 m KOH (Figure 10c). Because of the mismatch between the irregular pore shapes, the randomly entangled BCNNTs cannot facilitate access to the electrolyte ions (Figure 10d). Superior electrochemical properties of VA‐BCNNTAs, as opposed to BCNNTs, result from greater ion diffusivity of VA‐BCNNTAs resulting from aligned pore architectures, resulting in a considerably larger capacitance than BCNNTAs^[^ ^118^ ^]^ with excellent cyclic stability.

Iyyamperumal et al. synthesized VA‐BCNNTAs over a Ni‐Fe‐coated SiO_2_/Si substrate using CVD approaches in another study. Here, VA‐BCNNTAs are synthesized using melamine diboride, a single compound containing nitrogen, carbon, and boron that helps simplify BCN nanotubes' growth without any post‐synthesis functionalization. They also found out that the length of BCNNT increases with increasing the pyrolysis temperature, and good alignment is observed at a temperature of 1000 °C. The oxidation of amorphous carbon layers that were concurrently co‐deposited above the BCN nanotubes at comparatively low deposition temperatures causes VA‐BCNNT to develop at 900 and 950 °C. In XRD, two significant peaks were found at 2θ values of 26.3 and 43.6^o^, which indicate (002) and (100) plane reflections, demonstrating the well‐graphitized behavior of the BCN nanotubes. The peak intensity at 26.3^o^ rose with increasing pyrolysis temperature, indicating that the VA‐BCN array grown at 1000 °C is higher quality than those developed at lower temperatures (Figure 10e). These vertically aligned BCNNTs were able to provide a specific capacitance of ≈312.0 F g^−1^ when compared to undoped multiwalled carbon nanotubes, having a value of 117.3 F g^−1^ and nonaligned BCN with a value of 167.3 F g^−1^.^[^ ^111^ ^]^ Following this, Yesilbag and group tried to enhance the specific capacitance of this VA‐BCNNTAs using TMD decoration,^[^ ^110^ ^]^ which was discussed in detail in the section BCN/TMDs hybrids.

Because of its great features, such as lightweight, low density, and porosity, aerogels are being evaluated as an ideal electrode material for supercapacitors. Even though academics have done much work in aerogels as supercapacitor electrodes in recent decades, the updating speed of information is getting faster and faster.^[^ ^137^ ^]^ Graphene aerogel (GA, 3D graphene) has received much attention for its ability to minimize graphene nanosheet aggregation. Because of the densely linked porous framework free of any binders or conductive agents, the internal resistance is low, and the ion diffusion rate increases. Utilizing the benefits of these GO aerogels and heteroatom doping, Liu et al., for the first time, reported a BCN‐based aerogel for supercapacitor applications (Figure 10f).^[^ ^131^ ^]^ **Table** **3** summarizes electrode architectures' effects on the performance of BCN‐based supercapacitors.

**Table 3:** Effect of electrode architectures on the performance of BCN‐based supercapacitors.

Electrode	Precursor and synthesis method	Architecture design	Specific capacitance	Capacitance retention and coulombic efficiency	Ref.
VA‐BCNNTAs	Hydrothermal followed by carbonization using CTAB over SiO_2_/Si substrate	Vertically aligned	547 F g^−1^	95% after 1000 cycles.	[118]
VA‐BCNNTAs	Ni‐Fe‐coated SiO_2_/Si substrate	Vertically aligned	321.0 F g^−1^	95% after 1000 cycles.	[111]

**Figure 11:**
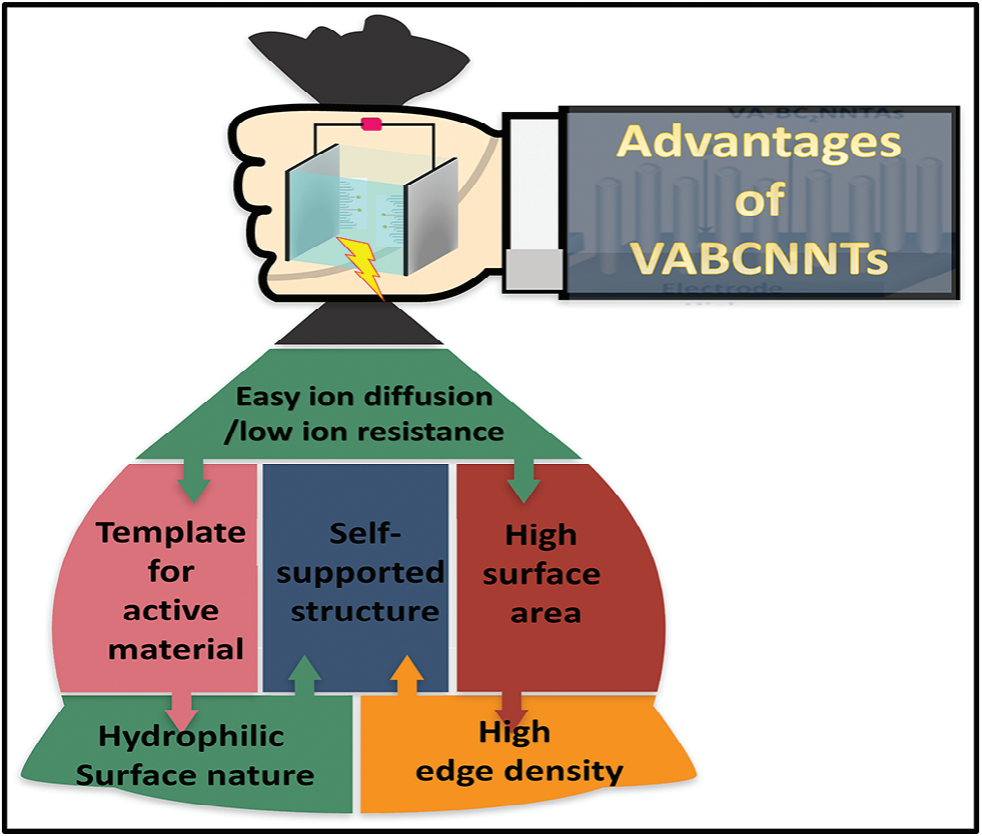
Advantages of VABCNNTs for supercapacitor applications.

Thus, compared with powder‐formed electrodes, these vertically aligned BCNNTs and aerogel provide benefits such as large surface area, a pathway for faster electron/ion transport, excellent mechanical properties, and intimate interface contact. The advantages of VABCNNTs are schematically represented in **Figure** **11**.

### 4.3 Effect of Electrolytes

Aqueous, ionic, organic, and solid‐state electrolytes are the four types of electrolytes. All of these have varying effects on the performance of supercapacitors. Organic electrolytes are made by dissolving salts such as TEABF_4_ in organic solvents such as acetonitrile or propylene carbonate. Organic electrolytes, when compared to aqueous electrolytes, can broaden the potential window of the supercapacitor. However, they are highly flammable, volatile, and toxic; their practical applicability has been limited and expensive. Poor specific capacitance and conductivity are two further issues that limit them. Even though organic solvents hold more salt than other solvents, acetonitrile harms the environment. Propylene carbonate, on the other hand, is environmentally beneficial. It offers a large voltage window across a wide temperature range. To achieve maximum specific capacitance, the electrode material's pore size should be proportional to the ion's size in the electrolyte, regardless of the electrolyte employed. Otherwise, the ions will be unable to reach the reaction sites. The electrolyte solution concentration must be increased to provide the capacitor's most significant achievable voltage output because even 3–5 ppm of water can significantly diminish voltage. The aqueous electrolyte might be neutral, acidic, or alkaline. Compared to organic electrolytes, they have a more significant ionic concentration and, as a result, a higher conductivity. However, their working potential is limited to near 1.2 V. In studies done by Panda et al., they already demonstrated that redox‐active N─ and O─ ‐functional groups make BCN better candidates for supercapacitor applications.^[^ ^120^ ^]^ The electron deficiency in the boron atom can attract more OH^−^ ions, producing good supercapacitive performance in aqueous and alkaline electrolytes. Their studies demonstrated that compared to 6.0 M potassium hydroxide (KOH), the KOH‐activated BCN performs better in acidic electrolytes such as 1 M sulfuric acid (H_2_SO_4_) Smaller cations such as (H^+^) have acidic electrolytes than larger (K^+^) ions having alkaline or neutral electrolytes. However, the large capacitance in the acidic electrolyte, such as H_2_SO_4_, is attributable primarily to the hydrogen storage mechanism.^[^ ^120^ ^]^ The performance of VABCNNTs in acidic and alkaline electrolytes was studied by Iyyamperumal et al. in acidic electrolytes 1.0 m H_2_SO_4_) and in alkaline electrolytes 6.0 m KOH. These VABCNNTs delivered a capacitance of 312.0 F g^−1^ in 1 m H_2_SO_4_ and 321 F g^−1^ in 6.0 m KOH. Here, the electrochemical performance in the basic medium is superior to that of the acidic electrolyte solution because the working potential in the acidic medium is substantially higher.^[^ ^111^ ^]^ The performance of supercapacitors can be enhanced by utilizing neutral electrolytes such as lithium nitrate (LiNO_3_), sodium sulfate (Na_2_SO_4_), sodium nitrate (NaNO_3_), lithium sulfate (Li_2_SO_4_), etc., because of enhanced adsorption of cations such as Li^+^ and Na^+^ at the electrode/electrolyte surfaces. This cation adsorption effectively reduces the hydrogen evolution reaction activity by further inhibiting the adsorption of H^+^ ions. Thus, neutral electrolyte helps to enhance the performance of aqueous supercapacitors by accumulating extra Na^+^/Li^+^ on the electrode surfaces. These charge accumulation layers tend to break down the build‐in‐electric field generated due to the heterostructure formations, thereby helping to enhance the diffusion/storage of Na^+^/Li^+^. Based on the above concept, MnO/MnS@BCN heterostructure was used for the construction of a supercapacitor with a sizeable potential window of ≈1.8 and 2 V in three‐electrode and two‐electrode setup in 1.0 m Li_2_SO_4_ with energy density ≈74.0 Wh kg^−1^.^[^ ^138^ ^]^

**Figure 12:**
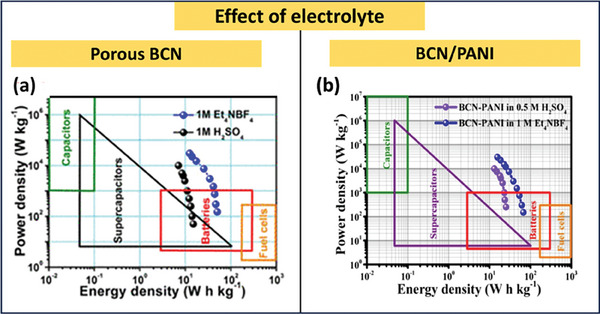
a) Performance comparison of Porous BCN in ionic liquid and acidic electrolyte (Reproduced with permission.^[^ ^114^ ^]^ 2023, American Chemical Society). b) Performance comparison of BCN/PANI in ionic liquid and acidic electrolyte (Reproduced with permission.^[^ ^139^ ^]^ 2023, Elsevier).

BCN‐based materials show better performance in ionic liquid electrolytes as well. Wang et al. demonstrated the performance of bandgap‐modified BCN in both 1.0 m H_2_SO_4_ and ionic liquid electrolytes such as 1.0 m tetraethylammonium tetrafluoroborate (Et_4_NBF_4_). This modified BCN shows a specific capacitance of ≈108 F g^−1^ at 0.1 A g^−1^ a with a maximum energy density of ≈14.8 Wh kg^−1^ and power density of ≈5.2 kW kg^−1^ with a potential window of 1 V. The potential window of fabricating a coin‐cell using Et_4_NBF_4_–propylene carbonate ionic liquid. This coin cell was able to extend the potential window up to 3 V with an energy density of ≈50.4 W h kg^−1^ and a power density of ≈3 kW kg^−1[^ ^114^ ^]^ (**Figure** **12**a). Similarly, using the BCN‐ polyaniline (PANI) hybrid, a symmetrical supercapacitor was constructed by Shi et al. Here, the ionic liquid Et_4_NBF_4_ was able to enhance the potential window up to 3 V and thereby increase energy density to 67.1 Wh kg^−1[^ ^139^ ^]^ (Figure 12b). The most recent solid electrolyte employed in flexible supercapacitor applications is polymer‐based. The energy density and stability of a polyvinyl alchol (PVA‐based KOH solid‐state electrolyte utilized in a supercapacitor are outstanding. The primary benefit of solid electrolytes is that they are required for all‐solid‐state supercapacitors with flexibility. The recent studies on microsupercapacitors based on BCN given previously underlined these statements. **Table** **4** summarizes the effect of electrolytes on the performance of BCN‐based supercapacitors.

**Table 4:** Effect of electrolytes on the supercapacitor based on BCN.

Electrode	Precursor and synthesis method	Electrolyte	Specific capacitance/Energy density and power density	Potential window	Capacitance retention and coulombic efficiency	Ref.
BCN	Hydrothermal → Carbonization → KOH activation	6.0 m KOH	332 F g^−1^	0 to −1 V	93% 10 000 cycles	[120]
		1.0 m H_2_SO_4_	406 F g^−1^	0 to 0.9 V	75% after 10 000 cycles	
VA‐BCNNTs	CVD	6.0 m KOH	321 F g^−1^	0 to −1.2 V	95% after 1000 cycles	[111]
		1.0 m H_2_SO_4_	312 F g^−1^	−0.4 to 1 V	98% after 1000 cycles	
MnO/MnS@BCN	Hydrothermal followed by annealing and sulfurization	Li_2_SO4	698.9 F g^−1^ at 0.5 A g^−1^	−0.8 to 1 V	95.2% after 10 000 cycles	[138]
Bandgap modified BCN	Annealing	1.0 m H_2_SO_4_	108 F g^−1^ at 0.1 A g^−1^	0 to 1 V.	97.2% after 10 000 cycles	[114]
		1.0 m Et_4_NBF_4_	40.4 F g^−1^ at 0.1 A g^−1^	0 to 3 V	‐	
BCN‐PANI	Annealing and electrodeposition	1.0 m H_2_SO_4_	180.2 F g^−1^ at 0.5 A g^−1^.	0 to1 V	89.6% after 10 000 cycles	[139]
		1.0 m Et_4_NBF_4_	53.6 F/g at 0.1 A/g	0 to 3 V	‐	

### 4.4 Effect of Concentration Of Boron

**Figure 13:**
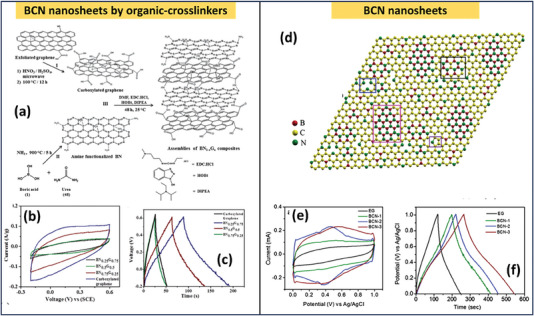
a) Schematic representation of BCN nanosheets produced by covalently cross‐linking graphene and boron nitride using EDC coupling, b,c) CV and GCD curves of BCN nanosheets synthesized using organic‐cross linkers by varying the Nitrogen and carbon ratio (Reproduced with permission.^[^ ^135^ ^]^ 2023, John Wiley and Sons). d) Schematic representation of BCN atomic model demonstrating incorporation of N and B in the graphene sheet, e,f) CV and GCD curves of BCN nanosheets, (Reproduced with permission.^[^ ^140^ ^]^ 2023 Elsevier).

The Zhou et al. studies demonstrated that the C, B, and N ratio in the VA‐BCNNTAs is ≈1:2:1, resulting in the stoichiometric ratio BC_2_N. The literature studies show that co‐doping porous carbon with B and N could enhance the specific capacitance because of the synergistic pseudocapacitive effect. The large amount of boron doping in the carbon layer with B/C is nearly ca.0.345, resulting in higher capacitance.^[^ ^118^ ^]^ Similarly, Ram Kumar et al. synthesized three different compositions of the BC_x_N_1– x_ composites (**Figure** **13**a) (*x* ≈ 0.25, 0.5, 0.75) by varying the B/N graphene ratio. Here, BC_0.25_N_0.75_ shows a capacitance of ≈180 F g^−1^, which also supports the studies done by Zhou et al. (Figure 13b,c).^[^ ^118^, ^135^ ^]^ However, studies by Panda et al. demonstrated that besides the ratio of boron and nitrogen, its activation with KOH and activation temperature significantly affects the performance of BCN‐based supercapacitors.^[^ ^120^ ^]^ In another study, a few‐layer BCN nanosheet using boric acid, urea, and few‐layer graphene at 900 °C was prepared by Sreedhara et al. Here, BCN sheets shown in Figure 13d with the least proportion of carbon and a large amount of nitrogen with stoichiometric ratio B_0.26_C_0.22_N_0.52_ show a specific capacitance of 306 F g^−1^ at 0.2 A g^−1^ shown in Figure 13e,f.^[^ ^140^ ^]^

## 5 Supercapacitor Application of BCN, Heteroatom‐Doped BCN, and BCN Hybrids

### 5.1 Pristine BCN

Chen et al. employed a pyrolysis technique to synthesize nanoporous BCN (1–50 µm) using a simple and green solvothermal method at a temperature of 250 °C. Here, sodium borohydride and 2,4,6‐tri(2‐pyridyl)‐triazine dissolved in acetonitrile and subjected to the solvothermal method. The product thus obtained was decomposed at different temperatures under the N_2_ atmosphere. Supercapacitive measurements are performed in the alkaline electrolyte of 6 m KOH. The rectangular‐like nature of BCN synthesized at 800 °C reveals the electrical double‐layer capacitive (EDLC) characteristics. These BCN sheets delivered a specific capacitance of ≈343.1 F g^−1^ at a current density of 0.5 A g^−1^. The charge/discharge test was repeated for 3000 cycles at a current density of 15 A g^−1^ to test the cycling life of the BCN electrode, and it showed a high capacitance retention of nearly 97.7%.^[^ ^141^ ^]^ Similar studies done by Chen and group BCN were prepared at a temperature of 800 °C. The specific capacitance (475 F g^−1^) and best cycle stability (97.7%) over 3000 cycles were demonstrated by this pristine BCN. This is due to BCN's higher specific surface area (506.9 m^2^ g^−1^), which can provide increased contact area and active site between the electrode material and electrolyte.

**Figure 14:**
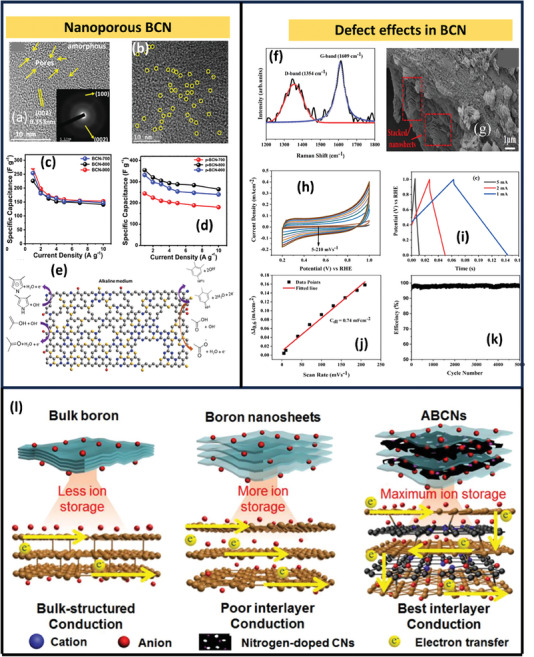
a,b) HRTEM images of porous BCN produced at 800 ^o^C by KOH activation, specific capacitance versus current density graph before, c) KOH activation, and d) after KOH activation, e) possible charge‐storage mechanism in KOH, (Reproduced with permission.^[^ ^120^ ^]^ 2023, American chemical Society). f) Raman spectrum showing defects, g) FESEM images of BCN nanosheets, h,i) CV and GCD curves of BCN nanosheets j) linear‐relationship showing peak‐current versus scan‐rate, k) efficiency after 5000 cycles, (Reproduced with permission.^[^ ^143^ ^]^ 2023, Elsevier). l) Electron and ion transfer in BCN electrode (Reproduced with permission.^[^ ^145^ ^]^ 2023, Wiley).

Similar to the previous study, Panda et al. synthesized 2D nanosheets identical to this approach where the precursors involved boric acid as boron source and formamide as N and C sources, and solvothermal carried out at a temperature of 220 °C. Here, the product obtained after pyrolysis was subjected to KOH activation under different temperatures ranging from 700 to 900 °C and helped to enhance the porosity, thereby increasing power density. This KOH activation happens as follows:

(1) $$2C+6\mathrm{KOH}\to 2K_{2}{\mathrm{CO}}_{3}+2K+3H_{2}$$

which can be further decomposed to produce CO_2_ and K_2_O at a temperature greater than 600 °C. Thus, the porosity of the composite was also found to increase (**Figure** **14**a,b). A considerable BET value is also observed for this KOH‐activated BCN, resulting in a high capacitive charge storage mechanism. The pore formation provides more defect sites, thereby increasing the electrolyte diffusion over the electrode material surfaces. Due to the presence of N, redox‐active O‐ and electron‐deficient B atoms, which can attract more OH^−^ anions, they exhibit a quasi‐rectangular trait representing the material's electrochemical double‐layer (EDLC) behavior. The specific capacitance calculated from CV for BCN before activation was ≈193.7 F g^−1^, and from GCD, this was ≈273 F g^−1^ with 85% capacitance contribution at a sweep rate of 60 mV s^−1^ (Figure 14c). After the KOH activation, it delivered a capacitance of ≈355 F g^−1^ at 1 A g^−1^ (Figure 14d). The high capacitance value can be due to the following: i) 2D nanosheets with enhanced porosity and large surface area. This reduces the electrolyte ion distances to access the interior regions of electrode material. Microspores with a size <1 nm result in pore confinement, enhancing the specific capacitance. ii) The heteroatom doping reduces charge transfer resistance and enhances capacitance. iii) The substitution of boron with carbon results in a downward shift in the Fermi level, thereby enhancing charge transfer and storage within this structure.^[^ ^120^ ^]^

The possible redox reactions in the KOH medium of this porous BCN are given in Figure 14e. Among the various approaches used for synthesizing BCN doping, in situ approaches, including direct carbonization, provide better performance.^[^ ^116^ ^]^ Thus, Li et al. synthesized BCN using a single‐step carbonization approach in the presence of ferric catalyst boric acid treatment after the composite formation between graphene oxide (GO) and waterborne polyurethane (WPU). This BCN‐based supercapacitor shows improved rate performance, high specific capacitance (330 F g^−1^ at 0.5 A g^−1^), and exceptional cycling stability. The fabricated symmetric supercapacitor using these BCN additionally demonstrated an excellent energy density (7.9 Wh kg^−1^) at 505 W kg^−1^ and a good capacitance retention of ≈89.9% in 6 m KOH electrolyte after 5000 charge–discharge cycles.^[^ ^142^ ^]^ The role of defects in the BCN sheets was studied by Kumar et al. The Raman spectra (Figure 14f) exhibit large D and G bands in the FWHM, which is indicative of structural imperfections where FESEM (Figure 14g) demonstrates a honeycomb‐like structure. The near‐rectangular CV curve (Figure 14h) in the potential window 0.2 to 1 V (Figure 14i,j) range demonstrates double‐layer capacitance properties with a non‐Faradic response between the surface of the electrode and electrolyte. The specific capacitance was ≈0.04 F g^−1^ at 1 mA. It shows excellent capacitance retention >90%, as shown in Figure 14k.^[^ ^143^ ^]^ Peng et al. utilized a template‐free CuBr_2_‐mediated carbonation of a cross‐linked boronate polymer for synthesizing porous BCN nanosheets. They discovered that the copper bromide (CuBr_2_) helps etch carbon skeleton but also reacts with carbon structure using redox reaction that helps to produce numerous pores. In 6 m KOH, this porous BCN was able to deliver a capacitance of nearly 258.5 F g^−1^ at 0.5 A g^−1^, and in the ionic liquid electrolyte 1‐ethyl‐3‐methylimididazolium tetrafluoroborate (EMIMBF_4_), the energy density was ≈43.7 Wh kg^−1^.^[^ ^144^ ^]^ To overcome the problems of boron nanosheets, such as low porosity and interlaminar conduction, Wu et al. synthesized anisotropic boron–carbon nanosheets using a gas‐phase exfoliation and condensation strategy. This helps to enhance the electrochemical properties and promote the smooth migration of electrolyte ions and excessive ion storage due to enhanced interlayer conductivity, accessible surfaces, and ionic pathways, as shown in Figure 14l.^[^ ^145^ ^]^ They also constructed a flexible supercapacitor using microfluidic spinning. **Table** **5** shows the performance of BCN‐based supercapacitors.

**Table 5:** Performance of BCN‐based supercapacitors.

Electrode	Precursor and synthesis method	Electrolyte	Specific capacitance/Energy density and power density	Capacitance retention and coulombic efficiency	Ref.
BCN	Boric acid and 2,4,6‐Tri(2‐pyridyl)−1,3,5‐triazine, Hydrothermal (250^o^ −24 h) followed by annealing	6.0 m KOH	343.1 F g^−1^ at 0.5 A g^−1^	90% stability after 3000 cycles	[141]
Porous BCN	Borohydride and 2,4,6‐Tri(2‐pyridyl)−1,3,5‐triazine, Hydrothermal (180°C‐24 h) followed by annealing	6.0 m KOH	475 F g^−1^ at 1 A g^−1^	97.7% after 3000 cycles	[116]
BCN	Formamide and boric acid, Hydrothermal 220 °C for 48 h followed by annealing and KOH activation	1.0 m H_2_SO_4_	355 F/g at 1 A/g	75% after 10 000 cycles	[120]
BCN	Waterborne polyurethane and GO carbonization	6.0 m KOH	330 F g^−1^ at 0.5 A g^−1^ Symmetric device ED/PD (7.9 Wh kg^−1^ at 505 W kg^−1^)	89.9% after 5000 cycles	[142]
BCN	Boric acid, acetone, and ammonia solution, Hydrothermal at 700 °C for 24 h	0.5 m H_2_SO_4_	0.04 F G^−1^ at 1 mV	‐	[143]
BCN	One‐step CuBr_2_‐mediated carbonation of a cross‐linked boronate polymer (FPA)	6 m KOH	258.5 F g^−1^ @ 0.5 A g^−1^ and in ionic liquid EMIMBF_4_ (43.7 Wh kg^−1^ under power density 400 W kg^−1^	97.4% after	[144]

### 5.2 BCN Hybrids

#### 5.2.1 BCN/TMD Hybrids

**Figure 15:**
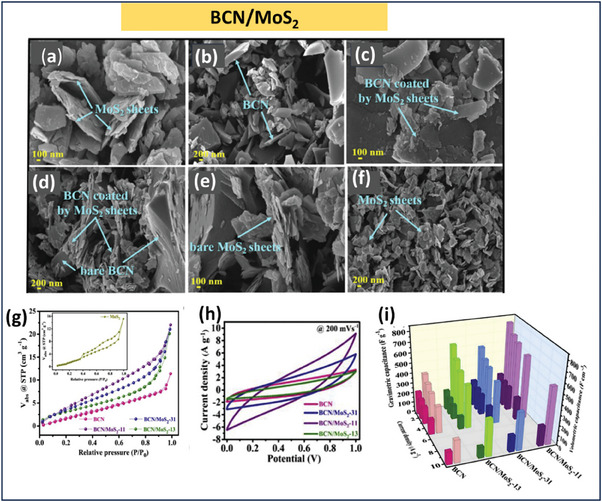
a–f) FESEM of MoS_2_/BCN. g) Adsorption‐desorption isotherm of MoS_2_/BCN. h) CV curves of BCN/MoS_2_, i) Ragone plot showing performance of MoS_2_/BCN, (Reproduced with permission.^[^ ^146^ ^]^ 2023, Elsevier).

In searching for high‐throughput and efficient BCN architectures, considerable attention was received by TMDs‐based BCN heterostructures, owing to the fascinating features studied in TMDs in advanced energy storage applications. TMDs in the form of MX_2_, where M is a transition metal, and X stands for chalcogens, often exhibit rich redox‐active sites with faradaic and capacitive contributions and exposed redox‐active surface areas with surface adjustable characteristics. However, there are several drawbacks, including insignificant chemical and microstructural stability, the rate of ion/charge transfer between the interfaces of electrode/electrolyte, minimal electronic conductivity, and inaccessible surface‐active sites, all of which could be critically solved by heterostructure formations. In this scenario, BCN invariably addresses the concerns regarding TMDs as a feasible heterostructure material for synergistic enhancements and problem suppression in high‐performance supercapacitor applications. Similar to other 2D/2D heterostructures having a facile electrostatic attraction, Thakur et al. reported a BCN/MoS_2_ heterostructure for the fabrication of supercapacitor applications, realizing the MoS_2_ with improved structural integrity and pseudocapacitive contribution of BCN. This hybrid formation was confirmed using XRD, and found that the inclusion of MoS_2_ helps to enhance the BCN crystallinity. This enhancement in crystallinity provides well‐accessible interfaces/surfaces, thereby improving electrochemical properties. FESEM also confirms the formation of this hybrid structure and found out that with an increase in the concentration of MoS2, they were agglomerated over BCN **Figure** **15**a–g. The excess amount of MoS_2_ can retard the porous nature of the hybrid and leads to a reduction in the surface area and thereby diminishing electrochemical performances. This hybrid exhibits a capacitance of ≈283 F g^−1^ (Figure 15h), and they are more electrochemically stable than BCN and MoS_2_. An enhanced power density (258 W kg^−1^) and energy density (9.8 Wh kg^−1^) (Figure 15i) can be seen for the BCN/MoS_2_ hybrid when compared to all other electrode materials.^[^ ^146^ ^]^ The adopted MoS_2_ synthesis strategy is typically based on solution reaction methods, and the resulting MoS_2_ generally appears in particle shapes, which not only results in a small specific surface area of the active electrode material but also makes effective interfacial contact between MoS_2_ and BCN.^[^ ^147^ ^]^

Thus, Tu et al. recently prepared BCN/MoS_2_ nanofibers using CVD and magnetron sputtering. The CVD method helps to fabricate BCN nanofibers directly over the carbon paper without any binding agent. In contrast, the magnetron sputtering helps activate the basal plane of MoS2, creating entrenched valleys and an expansion of active edge sites. The BCN fiber fabricated using CVD possesses excellent electrical conductivity, high surface area, and superior cyclic stability. This sputtering over BCN helps to prevent the aggregation of MoS_2,_ resulting in increased surface area. Second, the MoS_2_ coated over BCN can effectively protect BCN fibers from corrosion, enhancing stability. The sp^2^‐C bond in the BCN can support the lone pair electron to the empty 3d orbital of the Mo atom in MoS_2_. The electrophilic nature of the B atom helps to improve the wetting property of electrodes. Thus, this material possessing high active surface area, good conductivity, excellent charge transfer rate, and good wetting properties is an ideal electrode material. Therefore, this material shows excellent capacitance of ≈446.3 Fg^−1^ at 0.25 A g^−1^ in 1 m KOH electrolyte with maximum power density and energy density values of ≈1.9 kW kg^−1^ and 33.3 Wh kg^−1^.^[^ ^148^ ^]^

Vertical standing arrays are constructed as nanostructured arrays that stand vertically on conductive substrates. Compared to typical powdered electrodes, the rational design of vertical‐standing arrays can improve electrochemical performance through in situ growth techniques. This performance enhancement can be observed in vertically grown BCN nanotubes synthesized over graphene using Fe catalyst with CVD. The capacitance of vertically grown BCN nanotubes (VABCNNT) over graphene is roughly 364 F g^−1^ at 0.5 A g^−1^, which is very high compared to 2D BCN. Yesilbag et al. showed that encapsulating these VA‐BCN‐NT with TMDs such as WS_2_ helps to double the performance. This nanotube promotes electron and ion transport while providing flexibility and capacitive contribution. It also has good conductivity for WS_2_ and can be used to construct 3D networks with a core‐shell structure. The B and N presence in the BCN makes it more functional and helps in the growth of WS_2_ to form a core‐shell structure. This effective supercapacitive performance is due to the synergistic impact of WS_2_ and BCN nanotubes in a 3D core‐shell configuration. They built a two‐electrode symmetric set‐up with a Whatman fiberglass separator, which has higher electrical insulation and lower resistance during electrolyte ion transfer. It also has more robust chemical stability, preventing physical electrode transmission. This symmetric arrangement delivered a specific capacitance of ≈147 F g^−1^ at 20 A g^−1^. The electrode retains 85% capacitance after 10 000 cycles, indicating excellent stability. The assembled supercapacitor device exhibited a power density of ≈4250 W kg^−1^ and an energy density of ≈23.6 Wh kg^−1[^ ^110^ ^]^ **Table** **6** summarizes the performance of supercapacitors based on BCN/TMDs hybrids.

**Table 6:** Performance of supercapacitors based on BCN/TMDs hybrids.

Electrode	Synthesis method	Electrolyte	Specific capacitance/Energy density and power density	Capacitance retention and coulombic efficiency	Ref.
BCN/MoS_2_	BCN/MoS_2,_ Hydrothermal integration	1.0 m H_2_SO_4_	283 F g^−1^ at 1 A g^−1^ 9.8 Wh kg^−1^/258 W kg^−1^)	92% after 5000 cycles	[146]
BCN/MoS_2_	CVD	1.0 m H_2_SO_4_	446.3 F g^−1^ 0.25 A g^−1^ 33.3 Wh kg^−1^/1.9 kW kg^−1^	91% after 5000 cycles	[148]
VA‐BCNNT decorated with WS_2_	Hydrothermal	6.0 m KOH	690F g^−1^ at 0.5 A g^−1^	74% after 10 000 cycles	[110]

#### 5.2.2 BCN‐MXene and BCN‐rGO‐Based Hybrids

BCN's hierarchical structures, as well as its hybridization with MXene, have increased electrochemical performance. BCN and MXene structures function as excellent conducting networks, offering a pathway for fast electron transport while reducing inherent resistance and enhancing reaction kinetics. Aside from this, the features of BCN, such as electronegativity, and MXene, such as numerous ridges and rough surfaces, contribute to improved electrochemical performance. BCN/MXene composite preparation was studied using annealing or direct pyrolysis. The first of this kind of report on BCN/MXene using hydrothermal and annealing was reported in 2021 for the application of supercapacitor by introducing MXene acts as a good conducting bridge for the fast transport of electrons. Including BCN also permitted the nanoscale current collection, increased electron transfer with the higher interfacial area, improved MXene surface utilization, realization as a flexible matrix, and alleviation of volumetric difference in charge storage, all of which enhanced electrochemical performance. Hence, wearable all‐solid‐state microsupercapacitors fabricated by Tu et al. by decorating 3D‐BCN microsphere over MXene sheets. These MXene/BCN microflower interlayers can effectively enhance the dispersion of MXene nanosheets, giving ample space for ion transport. Second, nitrogen and boron atoms with varying electronegativities introduced into MXene nanosheets breach the MXene electroneutrality to produce active sites for electrochemical energy storage. The high H^+^ binding energies of the N‐5 and N‐6 planes of MXene/BCN boost the pseudocapacitance. By developing holes in the MXene/BCN structure, the electron‐rich nitrogen atoms in the compound materials can increase electron introduction and accelerate electrolyte wetting, substantially facilitating ion transfer and generating a double‐layer structure. Furthermore, the defects, good dispersion, and grain boundaries formed in the MXene/BCN microflower heterostructure function as excellent redox centers for charge separation, contributing to the increase in pseudocapacitance.

**Figure 16:**
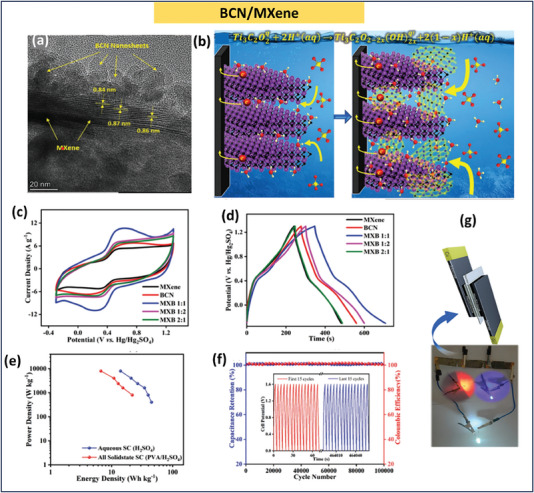
a) HRTEM Image showing heterostructure formation of BCN/MXene, b) Charge storage mechanism in 2D/2D MXene/BCN hybrids, c) CV curves of BCN/MXene hybrids, BCN and MXene, d) GCD curves of BCN/MXene hybrids, BCN and MXene, e) ragone plot and (f) capacitance retention after 100000 cycles g) Lightening LED using fabricated symmetric device, (Reproduced with permission.^[^ ^132^ ^]^ Copyright 2022, Wiley‐VCH.)

Understanding the significance of in‐situ composite material formation, Nasrin et al. synthesized MXene/BCN (MXB) using direct pyrolysis for supercapacitor applications. This strategy helps grow BCN over MXene nanosheets, forming 2D/2D interconnected networks. Benign reagents such as melamine, boric acid, and MXene were subjected to direct pyrolysis at elevated temperatures. This removes many oxygen‐contained termination groups from the Ti atom's surfaces, significantly increasing the energy storage performance via faradaic reaction. High‐temperature pyrolysis allowed for the in‐situ development and manufacture of BCN nanosheets all over the surface and perimeter of MXene, resulting in the formation of heterostructures. The hybrid formation was validated using HRTEM (**Figure** **16**a), which shows that a large amount of profuse BCN nanosheets grown over the MXene sheet, with appreciable nanosheets sticking at the periphery, enhancing the highly intriguing electrochemical properties of the heterostructure. MXB heterostructure performs better due to i) the redox mechanism as well as double‐layer capacitance due to the hydronium ion exchange and surface group bonding/debonding interactions occurring in MXene, ii) the pyrolysis will expose the Ti atom of MXene due to fraction elimination of terminal groups a where the molecules such as O_2_ and H_2_O will provoke the exposed Ti atom redox activity and lastly iii) the redox nature of BCN (Figure 16b). The MXene concentration greatly influences the specific capacitance, whereas the BCN and MXene with 1:1 concentration show better performance, which can be visible in (Figure 16c,d). This faradic behavior at interfaces, as well as the synergistic and individual contributions from BCN and MXene, results in increased capacitance, power density (14 Wh kg^−1^), and energy density (8000 Wh kg^−1^) with excellent stability (Figure 16e,f) in the symmetric device, as well as the ability to light LED (Figure 16g). This study proves that synergistic 2D/2D heterostructure is a feasible solution for integrating with efficient energy storage systems such as miniaturized electronics.^[^ ^132^ ^]^ **Table** **7** summarizes the performance of BCN/MXene‐based hybrids. Even while titanium‐based compounds (Ti_3_C_2_T_x_ and Ti_2_CT_x_) hybrids have been extensively researched for supercapacitor applications. Nb_2_C has garnered relatively less attention in the research community over the years owing to the complicated and sophisticated nature of its synthesis techniques. In contrast, theoretical predictions indicate that Nb_2_C has a higher areal and gravimetric capacitance than the extensively researched Ti_3_C_2_T_x_ MXene, underlining its potential as a promising option for advanced energy storage applications, considering these and excellent properties of 2D/2D MXene hybrids for supercapacitor applications Nasrin and group synthesized Nb_2_C/BCN hybrid structure. They used a hydrothermal method, and during this approach, Nb_2_C will partially dispart into 1D Nb_2_C structures and decorate over 2D Nb_2_C MXene sheets. Here, the Ostwald ripening drives the growth of 1D Nb_2_C nanorods, where 2D Nb_2_C MXene nanosheets act as a breeding ground for the uniform growth of 1D Nb_2_C nanorods. Later, using the pyrolysis approach, ultra‐small petals, similar to BCN sheets, were developed across the surface of 1D‐2D Nb_2_C. The 1D‐2D/2D Nb_2_C/BCN material exhibits exceptional electrochemical properties. It has an excellent specific capacity of 765 F g^−1^ at a current density of 2 A g^−1^, which can be attributed to its excellent charge storage performance at the interface. In symmetrical aqueous configurations, the NBCN electrodes provide remarkable energy density (35 Wh kg^−1^) and a high‐power density (3050 W kg^−1^). Furthermore, because of its large voltage window and the stability of its hybrid structure, the supercapacitor has excellent cycling stability.^[^ ^149^ ^]^ Another 3D structure combing 2D Ti_3_C_2_T_x_ and 1D BCN nanotubes using vacuum filtration in a sandwich and mixed method was synthesized by Yesilbag et al. Compared to 2D structures, this 1D BCN provides a larger surface area and more active sites for the adsorption and desorption of ions. The dopant atoms' (B and N) presence aids in developing more active sites and further enhances the pseudocapacitive behavior. This 2D Ti_3_C_2_T_x_/1D BCN could deliver a capacitance of nearly 678 F g^−1^ at 0.5 A g^−1^ because of the homogenous distribution of BCN nanotubes and exhibited excellent stability. Compared to a mixed structure, the sandwich structure of BCN/MXene provides a lower capacitance value. This may be because the electrolyte ions can't diffuse much more quickly in the sandwich structure. On the other hand, in a mixed structure, the BCN nanosheets help to prevent the restacking of MXene nanosheets and provide more facility for ion diffusion into the structure. Thus, this sandwich structure of BCN/MXene could deliver a capacitance of nearly 249 F g^−1^ only.^[^ ^150^ ^]^

**Table 7:** Supercapacitor performance of BCN/MXene‐based hybrid

Electrode	Synthesis method	Electrolyte	Specific capacitance/Energy density and power density	Capacitance retention and coulombic efficiency	Ref.
BCN/MXene	Direct pyrolysis	1.0 m H_2_SO_4_	1173 F g^−1^ (1876 C g^−1^) at 2 A g^−1^ Solid‐state device‐22 Wh kg^−1^	The solid‐state device shows 100% after 100 000 cycles	[132]
BCN/MXene	BCN was synthesized using the floating‐catalyzed chemical vapor deposition (FCCVD) method and BCN/MXene heterostructure using vacuum filtration	1.0 m Na_2_SO_4_	In a three‐electrode system, 678 F g^−1^ at 0.5 A g^−1^ and in the device. Symmetric supercapacitor‐ 160 F g^−1^ at 0.5 A g^−1^, energy/power density (32 Wh kg^−1^/11.5 kW kg^−1^)	97% after 10 000 cycles.	[150]
Nb_2_C/BCN	Hydrolysis +pyrolysis	1.0 m H_2_SO_4_	765 F g^−1^ at 2A g^−1^ in three‐electrode and solid‐state device 140 F g^−1^ at 0.5 A g^−1^ with energy and power density ≈13 Wh kg^−1^ and 276 W kg^−1^	100% retention after 10 000 cycles	[149]

As a highly researched material, reduced graphene oxide (rGO) or graphene has exploded in energy storage, particularly supercapacitors, with enormous commercial potential. Because graphene contains oxygen functional groups, it is a good foundation for coordinating with complementing elements to make composites. The rGO‐based hybrids were usually prepared by electrostatic interaction/self‐assembly or by simple physical mixing. The first kind of such report on BCN/rGO was investigated in 2022. This hybrid formation with BCN helps prevent the aggregation of rGO and reduces the structural rigidity of BCN microspheres.

#### 5.2.3 BCN‐Polymer‐Based Hybrids

**Figure 17:**
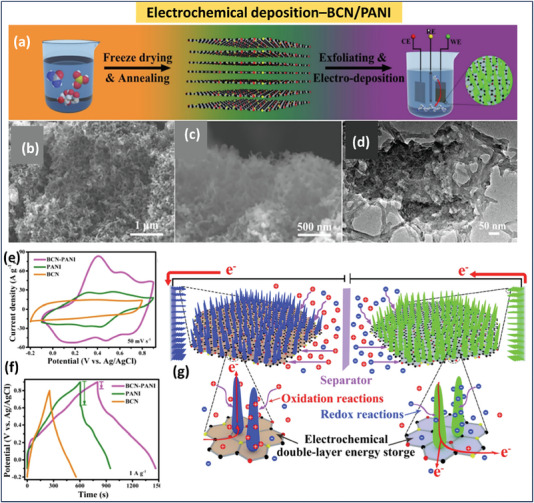
a) Schematics showing synthesis of BCN‐PANI composite, b–d) morphology of BCN‐PANI studied using FESEM and TEM e,f) CV and GCD diagram of BCN/PANI composites obtained in three‐electrode system in acidic electrolyte, g) Charge transfer mechanism (Reproduced with permission.^[^ ^139^ ^]^ 2023, Elsevier).

Nanohybrid conducting polymers (NHCPs) are a unique class of materials produced by utilizing the suitable method to combine two or more different types of nano moieties. The resulting NHCPs have distinctive physical features and are widely used in various industries, including producing batteries, supercapacitors, microelectronics, and sensors.PANI has the highest theoretical capacitance of any known conducting polymer due to its multiple redox states. PANI has received much attention recently because of its peculiar electrical properties, facile dopant doping/release energy storage strategies, and active pseudocapacitive behavior caused by quick and reversible Faradic reactions. However, due to multiple recurring volume changes in the polymer chain during charging and discharging, PANI has a limited cycle life, significantly limiting its utility in energy storage devices. As a result, carbon materials such as graphene are frequently utilized as substrates to house the active polymer, providing structural support and improving the cyclic stability of pure conducting polymers. However, giving flaw anchors when synthesizing electrochemically functional materials is problematic because pure carbon compounds are often non‐polar. At this point, improving PANI cycle stability by optimizing BCN's energy storage behavior by PANI modification and using defect‐enriched BCN as a growth substrate is a logical fit. Herein, Shi et al. modified BCN nanosheets with PANI using a simple electrodeposition method (**Figure** **17**a). They exfoliated BCN using a ball‐mill approach and obtained BCN nanosheets to anchor the PANI to get a hybrid. The capacitance of this exfoliated BCN‐PANI is ≈1360.5 F g^−1^ at 0.15 A g^−1^, which is 1.8 times greater than that of bulk BCN‐PANI. Here, CV techniques were utilized, in which polyaniline deposition begins with the formation of positive ion radicals of monomer aniline by aniline oxidation, then oxidizes on the surface of the electrode to form oligomers and subsequently autocatalyzes to produce polymers. As the number of CV depositions ascends, more PANI is gradually deposited on the BCN surfaces. The PANI morphology also changes, transitioning from particles with diameters <10 nm to nanorods, as shown in Figure 17b–d. PANI formation over the BCN surface was uneven without exfoliation, with many agglomerated PANIs emerging from the BCN surface. As a result, the exfoliation procedure reduces electroactive element accumulation and assists the BCN current collector in making good contact with PANI. This hybrid formation also conformed with XRD and Raman spectra. This composite shows an enlarged integration area in CV Figure 17e due to the advantageous conductive substrate that BCN nanosheets provide and the special synergy effect that may hasten electron transfer and speed up the electrochemical kinetic process. This composite was able to deliver a capacitance of ≈1360.5 F g^−1^ at 0.15 A g^−1^ (Figure 17f), whereas the bulk BCN/PANI demonstrated a capacitance of ≈751.5 F g^−1^ at 0.1 A g^−1^ with very low‐rate performance. Furthermore, the specific capacitance of symmetric devices (130.4 F g^−1^) can retain capacitance retention of ≈89.6% even after 10 000 cycles, proving the material's outstanding cycling stability. Figure 17g depicts the double‐layer formation/redox reaction during charging/discharging.

Another conducting polymer, polycarbazole (PCz), has gained much attention in supercapacitors because of its porous nature, lightweight, and good electrical pseudocapacitive nature. But still, compared to other conducting polymers such as PANI, polypyrrole, polythiophene, etc., it possesses low conductivity and electrochemical stability. Thus, hybrid formation was a feasible strategy to overcome these issues. Like Panda et al., they prepared chemically activated BCN by KOH activation and designed a hybrid with polycarbazole using an in‐situ oxidative polymerization. The CV studies of PCz show a pair of redox peaks, which indicate the pseudocapacitive nature of the material. But the CV curves after incorporating BCN into PCz the hybrid exhibited a quasi‐rectangular, slim leaf, rectangular‐like shape, demonstrating the excellent charge transfer at the interface of electrode/electrolyte. The presence of carboxyl and hydroxide groups in the porous BCN sheets, which cause the loop to distort as the scan rate increases, could explain the CV curve's peculiar shape. As a result, the loop is smaller and has an oblique angle. Here in GCD, the coulombic efficiency at lower current density is lower for PCz, PCz/BCN‐30 wt.%, and PCz/BCN‐70 wt.% hybrid, which is ascribed to due to incomplete ion deintercalation, which could be connected to the material's inherent properties. Among this, PCz/BCN‐50 wt.% shows a large CV curve with a capacitance of nearly 196 F g^−1^ at 0.2 V s^−1^. With the increase in BCN concentration, stacking may occur, which diminishes the conductivity. Also, the aggregation of BCN nanosheets inhibits the redox property. This also hinders the surface transfer of electrons and ions in the electrolyte/electrode interfaces and electrode. However, only 800 cycles of cyclic stability testing were performed. As a result, the cyclic stability of this hybrid material still raises some doubts.^[^ ^151^ ^]^ **Table** **8** shows the performance of polymer/BCN‐based hybrids for supercapacitor applications.

**Table 8:** Supercapacitor performance of BCN/polymer‐based hybrid.

Electrode	Synthesis method	Electrolyte	Specific capacitance/Energy density and power density	Capacitance retention and coulombic efficiency	Ref.
BCN/PANI	Hybrid formation using electrochemical deposition	1.0 m H_2_SO_4_	1360.5 F g^−1^ at 0.15 A g^−1^ 180 F g^−1^ at 0.5 A g^−1^ with 25.3 Wh g^−1^ 10 kW kg^−1^	89.6% after 10 000 cycles	[139]
polycarbazole (PCz)/BCN	The in‐situ chemical oxidative polymerization method	3.0 m KOH	134 F g^−1^ at 3 mA g^−1^	‐	[151]

#### 5.2.4 Ternary Hybrids

**Figure 18:**
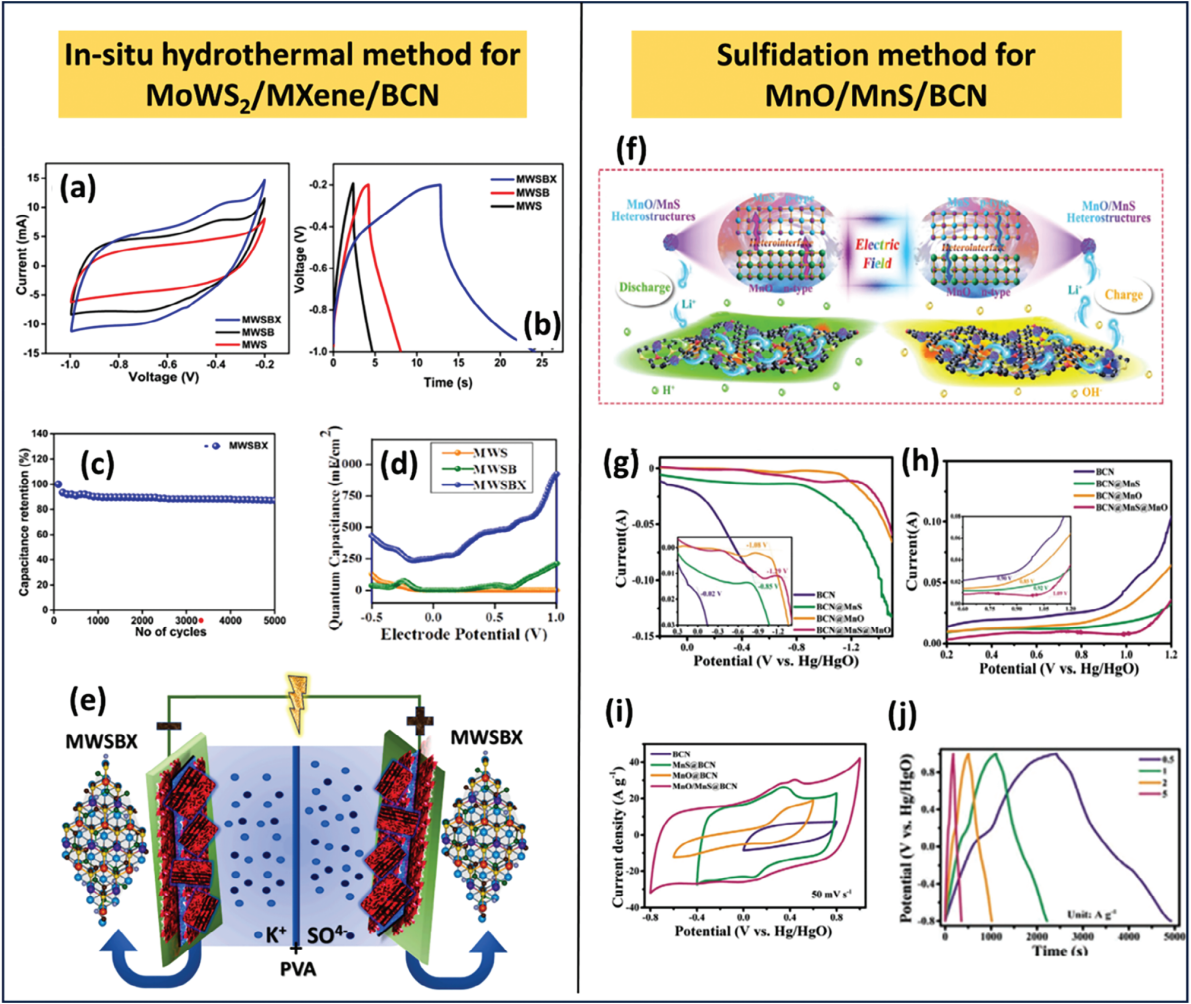
a) CV curves of MoWS_2_@MXene/BCN, MoWS_2_@MXene and MoWS_2_/BCN, b) GCD diagram showing performance of MoWS_2_@MXene/BCN, MoWS_2_@MXene and MoWS_2_/BCN, c) Cyclic stability after 5000 cycles, d) Quantum capacitance variation, e) Schematic representation of flexible SSD based on MoWS_2_@MXene/BCN (Reproduced with permission.^[^ ^152^ ^]^ 2023, Elsevier). f) Schematic representation of BCN/MnO/MnS charge‐transfer process, g) Polarization curves showing HER performance, h) Polarization curves showing OER performances, i) CV curves showing enhanced performance of BCN/MnO/MnS system, j) GCD curves showing enhanced performance of BCN/MnO/MnS system, (Reproduced with permission.^[^ ^138^ ^]^ 2023, John Wiley and Sons).

Ternary composites have recently gained popularity since two materials (binary composites) cannot match the specifications of a high‐performance supercapacitor. As a result, several methods for creating ternary composites for high‐performance supercapacitor applications have been developed by combining three different types of electroactive materials. Abhinandan et al. reported a ternary hybrid using MoWS_2_@MXene/BCN for the supercapacitor applications. Here, MXene and BCN act as a substrate for the growth of MoWS_2_ nanoflowers. This ternary hybrid formation helps to increase the surface area and interlayer separation, providing a higher number of active sites for the interactions and greater space for electrolyte ion transfer. Including BCN, followed by MXene in MWS_2_, they have demonstrated exclusive conductivity and greater charge transfer kinetics in the binary (MWS_2_/BCN) and ternary (MWS_2_/BCN/MXene) electrodes. All the CV curves in the three‐electrode system demonstrated a pseudocapacitive tendency, evident from the perceptible redox peaks (**Figure** **18**a). Again, the pseudocapacitive behavior of the samples has been verified using GCD profiles. The capacitive contribution for this ternary system was calculated using power law, which was ≈96%, representing the electrode's capacitive trait. An all‐solid‐state flexible symmetric device was fabricated to demonstrate the performance of this material. The CV curves revealed that MWS_2_/BCN/MXene had pseudocapacitive behavior, and the outstanding reversibility and rate capability were visible throughout all scan rates. From 10 mV s^−1^, the most significant areal capacitance calculated was 78 mF/cm^2^. The specific capacitance calculated was ≈289 mF cm^−2^ at 0.6 mA cm^−2^ (Figure 18b) with energy and power density ≈19.73 µWh cm^−2^ and 538.09 µW cm^−2^. This device also shows capacitance retention of ≈91% with 95% Coulombic efficiency (Figure 18c). MXene and BCN serve as templates for forming MoWS_2_ nanosheets, and the beneficial synergistic effect significantly increased the charge transit activity at the electrode‐electrolyte interface (Figure 18d). Thus, MWS_2_/BCN/MXene (Figure 18e) is a useful ternary hybrid electrode for supercapacitor application due to its abundant active sites, minimum ion diffusion, excellent charge allocation kinetics, resistance‐free channel for ion transfer, and better conductivity.^[^ ^152^ ^]^

MnS, a p‐type semiconductor, can be synthesized from MnO in a single‐step sulfurization method, yielding a binary hybrid containing MnO/MnS. Using this strategy, Shi and colleagues used GO as the precursor to create a ternary hybrid containing BCN and MnO/MnS, making a heterostructure that helps to increase the energy of adsorption for MnO/MnS for Li^+^ ion to −2.35 eV, which is higher than the individual components MnS and MnO. During the discharge procedure, a built‐in electric field arises between MnO and MnS due to the strong interaction between Mn and N atoms in BCN. As a result, Li^+^ ions can be entirely stored in MnO/MnS particles on the surface of BCN via the MnO‐MnS‐BCN pathway (Figure 18f). As a result, the electrode surface is positively charged and results in high electrostatic repulsion to H^+^ ions in the solution. This H^+^ ion migration inhibits hydrogen evolution reaction and raises the overpotential for hydrogen evolution (Figure 18g). During charging, two activation centers emerge over the MnO/MnS and BCN surfaces, increasing Li^+^ ion migration and causing separation from the electrode interface. Sulphide is more reversible in the p‐n heterostructure, driving a potential difference during discharge and reversing the electric field, i.e., from MnS → MnO. This spontaneously formed electric field can accelerate the separation of Li^+^ ions. Similarly, more Li+ ions are adsorbed on the BCN surfaces near the N site, resulting in an electric field from N to B. Also, there is a strong interaction between B and MnO/MnS. Thus, a proton transfer path from BCN (N site → B site) → MnS → MnO is possible, which will accelerate the migration of Li^+^ away from the electrode interface. Thus, during charging, SO_4_^2‐^ adsorbed at the surface while Li^+^ separated from the electrode's negatively charged interface, leading to the repulsion of OH^−^ ions and thereby increasing oxygen evolution potential (Figure 18h). In the end, the combined action of the charging and discharging process can increase the working voltage of the electrode material. Here, they found out that with an increase in MnS concentration, the capacitance of this ternary system increased until the molar ratio of MnS to MnO reached 1:8.6. This proposed ternary hybrid was able to deliver capacitance of nearly 698.9 F g^−1^ at 0.5 A g^−1^ and (Figure 18i,j) shows excellent efficiency even at lower current density. The Coulombic efficiency was ≈95.2% after 10 000 cycles. The symmetric device constructed using the same ternary shows a capacitance of about 347.9 F g^−1^ at a current density of 1 A g^−1^ with a potential window of 2 V with a maximum energy density of 75 Wh kg^−1^.^[^ ^138^ ^]^ **Table** **9** shows the performance of ternary hybrids based on BCN for supercapacitor applications.

**Table 9:** Performance of ternary hybrids based on BCN for supercapacitor applications.

Electrode	Synthesis method	Electrolyte	Specific capacitance/Energy density and power density	Capacitance retention and coulombic efficiency	Ref.
MoWS_2_@BCN@Ti_3_C_2_T_X_	Hydrothermal method	0.5 m K_2_SO_4_	289 mF cm^−2^ at 0.6 mA cm^−2^	95% after 5000 cycles	[153]
MnO‐MnS‐BCN	Sulfidation method	1.0 m Li_2_SO_4_	698.9 F g^−1^ at 0.5 A g^−1^	95.2% after 10 000 cycles	[138]

#### 5.2.5 BCN‐Based Asymmetric Supercapacitor

BCN is a better choice for fabricating asymmetric supercapacitors due to the previously discussed properties. Considering its excellent performance as a negative electrode from our group, Sreeraj et al. reported an asymmetric supercapacitor using S‐VSe_2_/CNT as the positive electrode and BCN as the negative electrode. Using the mass balance equation and forming the three‐electrode data, the mass ratio was ≈0.64. The fabricated asymmetric supercapacitor shows a potential window from 0 to 1.6 V. The constructed device exhibited a high capacitance of ≈96 mF cm^−2^ at a current density of 4 mA cm^−2^. ASC achieved a high energy density/power density of 36.3 mW h cm^−2^/3.2 mW cm^−2^ with 87% capacitance retention and 96% Coulombic efficiency.^[^ ^36^ ^]^

### 5.3 Doping of BCN

Doping is an essential strategy for improving the performance of pure nanomaterials. Doping aids in developing charged sites, which can be solid and active sites for electrochemical reactions. The doping helps to change the electronic structure of each other and thereby improves the electrochemical activity.^[^ ^154^ ^]^ For the first time, Liu et al. prepared a fluorine (F) doped‐BCN porous BCN aerogel for supercapacitor application. This F doping helps promote the charge redistribution between adjacent carbon atoms and produces high electronegativity, enhancing BCN's electrochemical stability and activity (**Table** **10**). They prepared this F‐doped BCN aerogel using graphene aerogel as the carbon substrate and ammonium tetra‐fluro borate (NH_4_BF_4_ as the F‐dopant. Doping these heteroatoms into the graphene aerogel helps to enhance the inter‐layer spacing, which means that these heteroatoms help in lattice distortions. The F‐doped BCN aerogel delivered a capacitance of nearly 529.4 F g^−1^ at 1 A g^−1^, indicating that co‐doping graphene aerogel with B, N, and F helps to enhance the electrochemical performances. The symmetric device can also deliver a capacitance of nearly 84.6 F g^−1^ at 0.1 A g^−1^ with a maximum energy density of ≈11.75 Wh kg^−1^. This strategy may open up an efficient method of co‐doping BCN to enhance performance.^[^ ^131^ ^]^

**Table 10:** Performance of doped BCN supercapacitors.

Electrode	Synthesis method	Electrolyte	Specific capacitance/Energy density and power density	Capacitance retention and coulombic efficiency	Ref.
Fluorine doped‐ BCN Aerogel	Hydrothermal followed by freeze drying and annealing	Aerogel	524.9 F g^−1^ at 1 A g^−1^	91.4% after 10 000 cycles	[131]

## 6 BCN‐Based Advanced Supercapacitor Devices

BCN‐based microsupercapacitors utilize a hybrid material called BCN to achieve high energy and power densities, making them ideal for compact electronic devices. Flexible supercapacitors offer flexibility and high power density, making them suitable for wearable electronics and bendable devices. Lithium‐ion supercapacitors combine the advantages of the energy density of lithium‐ion batteries with the rapid charge–discharge capabilities of supercapacitors, making them ideal for electric vehicles and renewable energy systems.

### 6.1 BCN‐Based Flexible Supercapacitors and Microsupercapacitors

**Figure 19:**
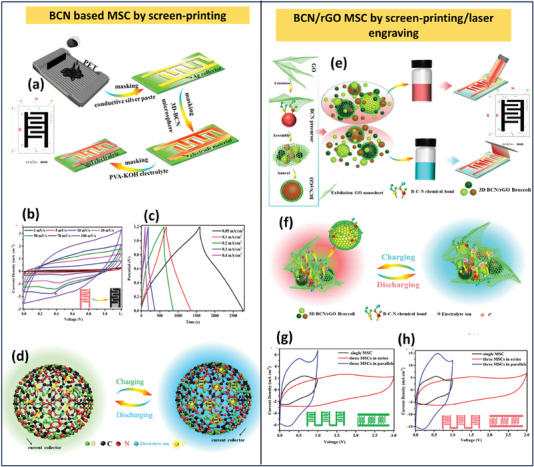
a) Schematic diagram showing fabrication of solid‐state flexible microsupercapacitors (MSCs) using 3D‐BCN microsphere, b,c) CV and GCD of MSCs based on 3D BCN microspheres, d) Charge‐storage mechanisms and performance, (Reproduced with permission.^[^ ^119^ ^]^ 2023, American Chemical Society). e) Schematic representation showing the fabrication of MSCs using BCN‐rGO hybrid, f) proposed mechanism of BCN‐rGO hybrid, g) CV test of MSCs BCN‐rGO hybrid array fabricated using screen‐printing at 30 mV s^−1^ in series and parallel combination, h) CV test of MSCs BCN‐rGO hybrid array fabricated using laser engraving at 30 mV s^−1^ in series and parallel combination, (Reproduced with permission.^[^ ^155^ ^]^ 2023, Elsevier).

Wireless and portable wearable electronics advancements demand a high energy density stable power supply. On‐chip energy storage integrated electrical devices became widely used to do this. Micro‐batteries were helpful to some extent, but micro‐supercapacitors can be a viable alternative when quick power delivery is necessary, and battery replacement is not an option. Most flexible microsupercapacitors have severe challenges with low energy density and poor cycle stability, which could be addressed by employing BCN electrodes. Tu et al. fabricated a micro supercapacitor utilizing the properties of 3D BCN microspheres using PVA‐KOH gel. They used screen printing (**Figure** **19**a) to fabricate all‐printed solid‐state flexible microsupercapacitors (MSCs) using these 3D‐BCN microspheres as electrodes. These MSCs based on 3D BCN exhibited an exceptional specific capacitance of 41.6 mF cm^−2^ at a current density of 0.05 mA cm^−2^ and an excellent cycle performance with good mechanical qualities (Figure 19b,c). Figure 19d shows that the N and B atoms, π electrons, open‐porous structure, and large surface area help enhance BCN microspheres' capacitance in MSCs. By absorbing electrons, the N atom facilitates double‐layer formation and ion transfer by generating holes in the C structure, thereby promoting electronic conductivity. Next, boron helps enhance wettability and capacitance via Faradic reactions. All these properties of BCN lead to high‐rate performance.

Due to their structural integrity, these BCN‐based microspheres were less acceptable for flexible devices. Thus, to address this issue, a standard supercapacitor material, graphene oxide (GO) nanosheet, was combined with BCN to make a flexible composite by Tu et al. The introduction of 3D BCN microspheres can reduce GO aggregation and contribute to the conversion of GO into rGO, allowing them to be readily anchored to structure, which indicates potential performance as flexible device electrode materials. The hybrid material based on 3D BCN/rGO broccoli was used in microsupercapacitors and fabricated using two approaches, i.e., screen‐printing and laser engraving (Figure 19e). BCN's 3D broccoli‐like structure (Figure 19f) resulted in a porous structure with a large surface area. Also, the N and B will break the carbon neutrality, resulting in faster ion diffusion and adsorption. The electrochemical performance of this composite was investigated using a screen‐printed electrode. This electrode could deliver a ≈72.2 mF cm^−2^ capacitance at 0.1 mA cm^−2^ current density. The CV curves of three such connected MSCs in series and parallel are given in Figure 19g. It demonstrates that the potential window of three MSCs in series increases three times that of a single MSC device, while the output current of three MSCs in parallel increases three times that of a single MSC device. The same hybrid was constructed using a laser‐engraving approach, and it could deliver a capacitance of ≈179 mF cm^−2^. As with screen printing, the three single MSCs in series tripled the voltage window, while three MSCs in parallel tripled particular capacitances, as illustrated in Figure 19h. This supercapacitor also shows excellent mechanical flexibility with a capacitance retention of ≈95%.^[^ ^155^ ^]^

Similarly, another microsupercapacitors utilizing the benefits of MXene/BCN micro flowers was reported by Tu et al., which was described earlier. These all‐solid‐state microsupercapacitors can reach an areal capacitance of ≈89 mF cm^−2^ with good cyclic retention. Overall, developing suitable electrode architecture and supercapacitor assembly is an exciting topic of energy research that can extract desired electrochemical properties.^[^ ^37^ ^]^ Among the various configurations in microsupercapacitors, interdigitated configuration will outperform because it facilitates the shortest distance between electrodes for ion transport. Among the various fabrication techniques of laser writing is a single‐step, faster technology that can be used to fabricate microsupercapacitors. For the first time, Karbhal et al., fabricated MSCs using BCN. At a current density of 0.15 mA cm^−2^, this BCN‐based microsupercapacitor demonstrated a comparatively very high specific capacitance of 72 mF cm^−2^. Even at a high current density of 1 mA cm^−2^, the device exhibited a specific capacitance of up to 17 mF cm^−2^. It has shown good electrochemical stability even after 80 000 cycles with no capacitance decay and good flexibility.^[^ ^123^ ^]^

**Figure 20:**
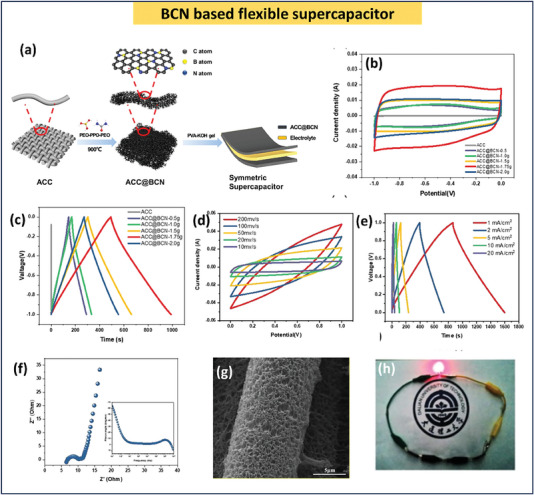
a) Schematic illustration demonstrating the construction of flexible MSCs based on ACC@BCN, b,c) Electrochemical performances of ACC and five kinds of ACC@BCN in three‐electrode system, d,e and f) Electrochemical performance of ACC@BCN based MSCs, f) EIS spectrum of ACC@BCN, g) Morphology after stability, h) LED‐lightening with constructed device, (Reproduced with permission.^[^ ^156^ ^]^ 2023, Elsevier).

Flexible all‐solid‐state supercapacitors are considered contenders for future energy storage devices due to their simple structure, extended cycle life, quick charging, and environmental friendliness. Nonetheless, flexible all‐solid‐state supercapacitors with high power density, high energy density, and stable cycle life at the same time remain extremely challenging, especially in the condition of multiple bending. As a result, fabricating electrode materials with high specific capacitance, flexibility, and cyclic stability is desirable. The literature study shows that BCN materials reported for supercapacitor applications are primarily powder. When BCN is bonded to the current collector as an electrode with a binding agent, the resistance increases dramatically, causing the specific capacitance to decrease. Electrode materials like nickel foam, carbon fiber cloth/paper, and aluminium foil can be grown directly on the current collector to boost performance. Shi et al. fabricated a flexible supercapacitor using a binder‐free approach where BCN was anchored on an active carbon fiber cloth (ACC) for the first time. This flexible supercapacitor was fabricated by immersing this ACC substrate in a solution containing urea, boric acid, and polyethylene oxide‐propylene oxide (P123) and subjected to calcination (**Figure** **20**a). Because of its layered porosity network, wide specific surface area, and high degree of heteroatom doping, ACC@BCN has a significant potential for application as an electrode material in fabricating flexible supercapacitors. The specific capacitance for this ACC@BCN in a three‐electrode system was ≈1018 mF cm^−2^ at the current density of 1 mA cm^−2^ (Figure 20b,c), which is the best value reported among the so far values. The ESR and Rct value is ≈1.41 and 2.52 Ω (Figure 20f), demonstrating a faster transfer rate in ion diffusion and good conductivity. It also retained a capacitance of ≈95.8% even after 10 000 cycles, indicating excellent stability of the electrode material. This 3D porous network structure remains unchanged even after 10 000 cycles, demonstrating the stability of the structure (Figure 20g). The symmetric cell constructed using PVA/KOH gel shows a working voltage window up to 1 V. The CV curves electrode exhibited a quasi‐rectangular shape, demonstrating the typical Faraday response of the electrode at different scan rates. This flexible symmetric device was able to deliver a capacitance of nearly 737.0 mF cm^−2^, which is the highest so far reported value. The red LED can be easily lightened by connecting two flexible devices in series (Figure 20h). This study describes a method for mass‐producing low‐cost, high‐performance flexible capacitor electrode materials for high‐performance energy storage devices.^[^ ^156^ ^]^

**Figure 21:**
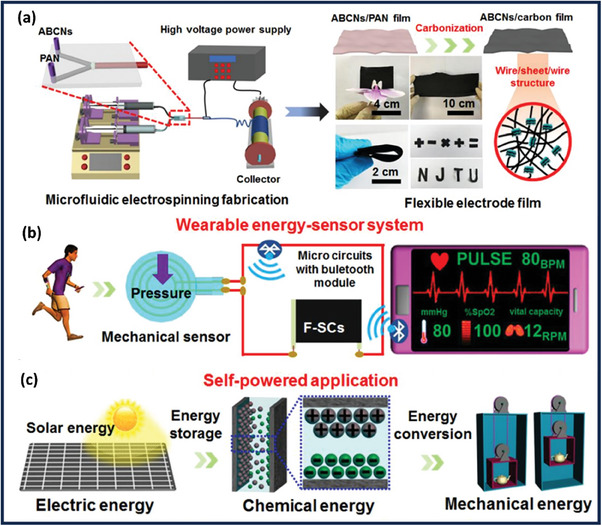
a) Schematic representation of BCN nanofibers prepared using microfluidic electrospinning with properties such as lightweight, high flexibility, large‐size, and easily tailoring properties, b) schematic illustration of the BCN‐based flexible supercapacitor integrated into the woven fabric with sensor and Bluetooth module. c) Schematic representation of self‐powered supercapacitor model, with permission.^[^ ^145^ ^]^ 2023, Wiley).

Microfluidic spinning is a new approach widely used to fabricate hybrid nanomaterials. This method facilitates generating a wide range of arrays and patterns with exceptional efficiency and scalability.^[^ ^157^, ^158^ ^]^ Chen et al. constructed a flexible supercapacitor based on BCN using this approach and MES technology, as shown in **Figure** **21**a. Here, solutions containing ABCNs/N, N‐dimethylformamide (DMF), and polyacrylonitrile were injected into microfluidic chips and subjected to carbonization. The film thus constructed was utilized for creating a flexible and wearable supercapacitor using poly(vinylidenefluoridehexafluoropropylene)/1‐eutyl‐3‐methylimidazolium tetrafluoroborate (PVDF‐HFP/EMIBF_4_) as electrolyte. This flexible capacitor was able to deliver a capacitance of nearly 534.5 F/cm^3^. Considering this flexible supercapacitor's impressive electrochemical performance, they used this system in a wearable energy sensor. In this wearable device, the BCN‐based flexible supercapacitor is used as a high energy supply to power a mechanical sensor, effectively monitoring human activities with consistent physiological information, as given in Figure 21b. In another practical use, the BCN‐based fiber supercapacitor and commercial solar cells are combined to create a self‐powered device. This self‐powered device may transform solar energy into electrical energy using a solar cell, in which a flexible supercapacitor can store electrochemical energy to power mechanical motions (Figure 21c).^[^ ^145^ ^]^ **Table** **11** shows the performance of BCN‐based microsupercapacitors and flexible supercapacitors.

**Table 11:** Performance of BCN‐based microsupercapacitors and flexible supercapacitors.

Electrode	Flexible/microsupercapacitors	Electrolyte	Specific capacitance/Energy density and power density	Capacitance retention and coulombic efficiency	Ref.
BCN/MXene	Microsupercapacitor by screen printing	PVA/KOH	41.6 mF cm^−2^ 0.05 mA cm^−2^ (0.00832 mW h cm^−2^/0.183 mW cm^−2^)	94% after 10 000 cycles	[37]
BCN	Microsupercapacitor using laser scribing	PVA/H_2_SO_4_	72 mF cm^−2^ at 0.15 mA cm^−2^	100% after 80 000 cycles	[119]
BCN	Flexible supercapacitor	PVA/KOH	737 mF cm^−2^ AT 1 mA cm^−2^ 1.207 mWh cm^−3^ 12.49 mW cm^−3^	95.8% after 10 000 cycles	[156]
BCN	Flexible supercapacitors using microfluidic electrospinning	(PVDF‐HFP/EMIBF_4_	534.5 F cm^−3^ at current density at 0.1 A cm^−3^, energy density of 167.05 mWh cm^−3^ at the power density of 0.15 W cm^−3^	94.1% after 10 000 cycles	[145]

### 6.2 Li‐Ion Supercapacitors

**Figure 22:**
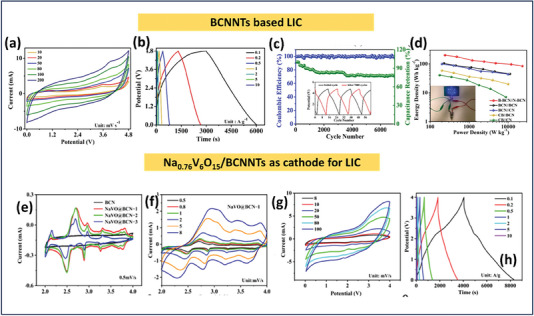
a,b) CV and GCD curves of the B‐BCN//N‐BCN LIC at various scan rates and current density, c) stability, d) Ragone plots of LICs based on BCNNTS and lighting LED panel, (Reproduced with permission.^[^ ^112^ ^]^ 2023, American Chemical Society). e) CV curves of the NaVO/BCNNT and BCNNTs, f) CV curve of NaVO/BCNNT at various scan rates, g) CV curves of the NaVO/BCNNT//HC based LIC, h) GCD curves of the NaVO/BCNNT//HC based LIC (Reproduced with permission.^[^ ^115^ ^]^ 2023 mdpi).

Lithium‐ion capacitors (LICs), which combine the benefits of supercapacitors and Lithium‐ion batteries, have recently received much interest. Because of the traits mentioned above, BCN is a preferable contender. LICs comprise a capacitive supercapacitor‐type cathode and a Faradaic lithium‐ion‐battery‐type anode with a lithium‐salt‐containing electrolyte. They have improved power density, energy density, and cycling stability due to diffusion‐controlled intercalation/deintercalation of lithium ions at the anode and physical adsorption and desorption processes at the cathode. However, due to the kinetic imbalance between the rapid adsorption and desorption of SC‐type cathodes and the gradual intercalation and deintercalation of lithium‐ion in LIB‐type anodes, it is very challenging for LICs to demonstrate an ideal power density, energy density, and good cycle life. BCN materials are accepted as suitable cathode materials for LICs because of their distinctive hollow tube structure, which may be exploited to create composite materials and ion transport channels. For the first time, Jiang et al. rationally designed LICs using B‐BCN as a cathode and N‐BCN as an anode. This was able to obtain a potential window of 0–4.8 V (**Figure** **22**a,b) with a higher energy density of ≈200 Wh kg^−1^ and excellent stability (Figure 22c,d).^[^ ^112^ ^]^

The performance of BCNNTS in LICs can be improved by decorating these BCNNTS with battery‐type cathode material. For example, Xu et al. decorated BCNNTS using battery‐type cathode material Na_0.76_V_6_O_15_ (NaVO) using a solid phase approach and hydrothermal method followed by annealing. During half‐cell characterization, BCNNTS shows rectangular CV, while the hybrid with NaVO shows three reduction peaks, representing the reduction of V^5+^ to V^4+^ and V^4+^ to V^3+^, respectively. This lithium‐ion intercalation and deintercalation process is as follows (Figure 22e,f):

(2) $${\mathrm{Na}}_{0.76}V_{6}O_{15}+x{\mathrm{Li}}^{+}+xe^{-}↔\mathrm{Li}x{\mathrm{Na}}_{0.76}V_{6}O_{15}$$

This NaVO/BCNNTS has the most significant specific capacity of ≈61.11 mAh g^−1^ at 1 A g^−1^. These studies show that the NaVO/BCN cathode is a superior cathode for high‐performance LICs because it combines capacitive and Faradaic behavior. They fabricated a full LIC using NaVO/BCNNTS as the cathode and HC as an anode. This constructed device shows a potential window ranging from 0–4 V (Figure 22g). The NaVO/BCNNTS/HC LIC's CV curves are rectangular in shape and feature several redox peaks, suggesting a synergistic mechanism took place in the device. The NaVO@BCNNTS cathode absorbed significant PF_6_ ions during charging (capacitive behavior), while Li^+^ was embedded in the HC anode (battery behavior). Additionally, the energy density was raised by the vanadium ions oxidation in the NaVO (battery behavior). The reverse process happens during discharging. This LIC shows a specific capacity of ≈107.43 F g^−1^ at 0.1 A g^−1^ (Figure 22h). With a good cycling life (65.5% after 5000 cycles), the constructed NaVO/BCNNTS/HC LIC offers a high energy density of 238.7 Wh kg^−1^ at 200 W kg^−1^.^[^ ^115^ ^]^

## 7 Critical Challenges and Future Outlooks

Incorporating BCN as a supercapacitor electrode material provides a slew of crucial issues that must be addressed meticulously for effective implementation. First and foremost, tailoring the characteristics and homogeneity of BCN demands precise modification of reaction conditions, precursor selection, and post‐treatment techniques. Reproducibility and scalability of BCN synthesis processes become critical for large‐scale manufacturing. Furthermore, while BCN has impressive electrochemical properties, its long‐term stability and cycling performance in supercapacitors require further improvement. Degradation processes, specific capacitance attenuation, and increased internal resistance over extended cycling all compromise the reliability and efficiency of BCN‐based electrodes. It is critical to unravel the various degradation mechanisms and devise counteractive measures to improve the durability and longevity of BCN‐incorporated supercapacitors. Furthermore, incorporating BCN electrodes into practical devices presents problems in device architecture and manufacturing. Integrating BCN electrodes and current collectors seamlessly, optimizing electrode thickness, and minimizing mechanical stresses and strains experienced during device operation are all critical issues. Innovative device designs and manufacturing procedures that overcome these hurdles become vital to successfully commercializing BCN‐infused supercapacitors. Finally, the high cost of BCN synthesis and the scarcity of precursor materials provide significant economic challenges. Scaling up BCN manufacturing while lowering production costs is essential to giving BCN‐based supercapacitors economic viability and promoting competition against existing energy storage technologies. Conquering these critical difficulties via committed research and development efforts is vital to realizing BCN's complete potential as a supercapacitor electrode material and announcing its practical implementation across various energy storage applications.

**Figure 23:**
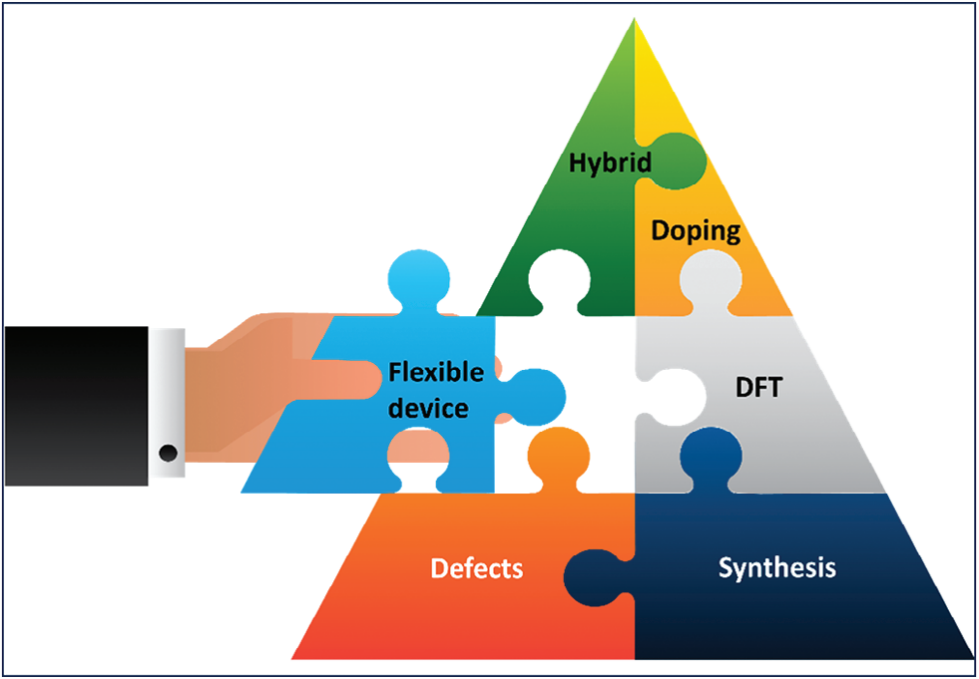
Future outlook of BCN‐based supercapacitors.

Various areas of attention in the future show significant potential for developing BCN‐based supercapacitor electrodes. One critical component is the investigation of more efficient and streamlined synthesis processes that avoid complex operations such as annealing and heat treatment, making supercapacitor device production easier. It is also critical to optimize the synthesis temperature and create evenly oriented procedures to produce greater yields of BCN in thin‐layer structures and enable large‐scale manufacturing. Furthermore, combining BCN with other compounds, such as conducting polymers, TMDs materials, and other layered materials, can considerably improve its electrochemical performance. More research in this area is required to leverage the potential of BCN‐based hybrids in supercapacitor applications fully. BCN's achieved charge storage capacity can be utilized to study real‐time supercapacitor applications. Exploration of heteroatom doping in BCN is also essential since it has shown promise in changing the material's characteristics in other 2D materials. Extensive research is necessary to comprehend the effect of heteroatom doping on the BCN network and its implications for supercapacitor performance. Because present knowledge in this domain is limited, theoretical research is also required to get deeper insights into BCN's fundamental characteristics and electrochemical processes. Furthermore, while creating flexible supercapacitor devices based on BCN electrodes is a promising trend, further study is required to explore the potential and overcome practical obstacles fully. Finally, flaw engineering, which has been used effectively in other 2D materials, still needs to be explored in BCN. Overall, these future views in synthesis methods, hybrid structures, heteroatom doping, theoretical understanding, flexible device manufacturing, and defect engineering will help to enhance and utilize BCN‐based supercapacitor electrodes. Investigating the impact of defect engineering on BCN may result in enhanced electrochemical performance and a broader range of applications. **Figure** **23** summarizes the future outlooks of BCN‐based supercapacitors.

## 8 Conclusion

This article provides a comprehensive review of BCN and its application in supercapacitors. The unique properties of BCN, including its structural, optical, magnetic, mechanical, and electronic characteristics, are extensively examined. Different synthesis methods such as chemical vapor deposition, solvothermal/hydrothermal method, template‐assisted synthesis method, and pyrolysis are discussed, supplemented by schematic diagrams. Additionally, the factors affecting the charge storage mechanism have been emphasized to encourage wide‐spreading BCN as a supercapacitor electrode. The article also explores the application of BCN as an electrode‐active material in supercapacitors, highlighting the use of nanocomposite/hybrid electrodes incorporating carbon nanomaterials, electronically conducting polymers, TMDs, MXene, and other materials. potential industrial applications of BCN‐based electrode‐active materials in lithium‐ion capacitors, microsupercapacitors, and other flexible devices are envisaged. In summation, using BCN as a supercapacitor electrode material showcases its immense potential and versatility, elucidating many distinctive attributes, an array of synthesis methodologies, and the scope for widespread industrial implementation in supercapacitor devices.

## Conflict of Interest

The authors declare no conflict of interest.
